# Curvature in the reproductive tract alters sperm–surface interactions

**DOI:** 10.1038/s41467-021-23773-x

**Published:** 2021-06-08

**Authors:** Mohammad Reza Raveshi, Melati S. Abdul Halim, Sagar N. Agnihotri, Moira K. O’Bryan, Adrian Neild, Reza Nosrati

**Affiliations:** 1 Department of Mechanical and Aerospace Engineering, Monash University, Clayton, VIC Australia; 2 IITB-Monash Research Academy, IIT Bombay, Mumbai, India; 3 School of Biological Sciences, Monash University, Clayton, VIC Australia; 4 School of BioSciences, Faculty of Science, University of Melbourne, Parkville, VIC Australia

**Keywords:** Microfluidics, Cellular motility, Microfluidics, Reproductive biology, Biological physics

## Abstract

The fallopian tube is lined with a highly complex folded epithelium surrounding a lumen that progressively narrows. To study the influence of this labyrinthine complexity on sperm behavior, we use droplet microfluidics to create soft curved interfaces over a range of curvatures corresponding to the in vivo environment. We reveal a dynamic response mechanism in sperm, switching from a progressive surface-aligned motility mode at low curvatures (larger droplets), to an aggressive surface-attacking mode at high curvatures (smaller droplets of <50 µm-radius). We show that sperm in the attacking mode swim ~33% slower, spend 1.66-fold longer at the interface and have a 66% lower beating amplitude than in the progressive mode. These findings demonstrate that surface curvature within the fallopian tube alters sperm motion from a faster surface aligned locomotion in distal regions to a prolonged physical contact with the epithelium near the site of fertilization, the latter being known to promote capacitation and fertilization competence.

## Abstract (web summary)

The effect of fallopian tube’s curvature on sperm motion has not been studied in detail. Here, the authors use droplet microfluidics to create soft curved interfaces, revealing a dynamic switch in sperm motility from a progressive surface-aligned mode at low curvatures, to an aggressive surface-attacking mode at high curvatures.

## Introduction

**Fig. 1:**
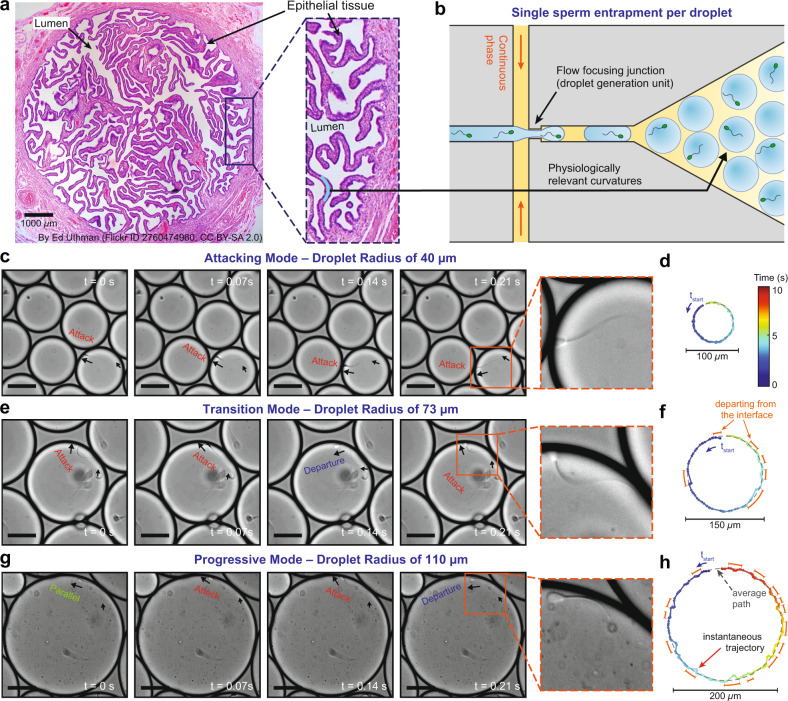
Sperm motility at curved interfaces representing the soft and folded epithelial tissue in the female fallopian tube. **a** The female fallopian tube is a complex microenvironment composed of soft and highly folded epithelial tissue, forming confined lumens with radius of curvatures ranging from ~20 μm to over 150 μm (refs. ^3,5,18^). **b** Encapsulation of individual sperm in monodisperse droplets ranging in size from 30 to 140 μm, mimicking the physiologically relevant range of curvatures in vivo. Time-lapse images and representative trajectories of sperm swimming in (**c**, **d**) aggressive attacking, (**e**, **f**) transition and (**g**, **h**) progressive surfaces aligned motility modes in 40, 73, and 110 µm-radius droplets, respectively. The color of the instantaneous swimming trajectories corresponds to time, as shown in the legend in (**d**). Orange dashed lines highlight the deviation between instantaneous trajectory and average path due to departing behavior. Scale bars, 50 µm. Images were contrast-adjusted for clarity. The fallopian tube cross-section in (**a**), entitled “Normal Fallopian Tube, Human”, by Ed Uthman is sourced without change from https://www.flickr.com/photos/euthman/2760474960/ under CC BY 2.0. Each experiment was repeated 5, 4 and 5 times independently with similar results for (**c**, **e**, and **g**), respectively. Source data are provided as a Source Data file.

Sperm migration through the female reproductive tract is crucial for fertilization^1–3^. The natural selection process in vivo is mainly achieved in the fallopian tube, where anatomical features and physiological conditions guide the sperm to the site of fertilization^1,4^. The fallopian tube is lined with complex labyrinthine epithelium which forms crevice-like lumens which narrow towards the egg^4–6^ (Fig. 1a) thus intensifying the role of hydrodynamic interactions in sperm function^1,4,7^. The interaction of spermatozoa with these complex epithelial surfaces is thought to provide geometrical guidance, increase the chance of survival^8–10^, trigger sperm attachment/detachment mechanisms^11^, stimulate capacitation^8^ (a physiological change in sperm to enable fertilization competence) and play a key role in the timing of fertilization^1,12^. It is known that chemical signaling, via oviductal secretory fluids^13–15^, and surface effects influence sperm motion^7,16–18^. However, how the physiology and anatomy of the soft curved epithelial surfaces in the fallopian tube regulates sperm dynamics and confers guidance is still poorly understood.

Sperm motion in vivo is influenced by confinement and surface proximity^3,19^, whereby hydrodynamic forces^20,21^, and steric repulsion^17,22^ cause the cell to follow boundaries and accumulate at interfaces^19,23,24^. Over the past 10 years, sperm motion and accumulation at flat solid surfaces have been studied extensively using advanced microscopy methods^7,25–27^, mathematical modeling^19,28–30^, and microfluidics^2,31–33^. The results have been an improved fundamental understanding of sperm locomotion, demonstrating new swimming modes^34^, and opportunities for high-quality sperm selection^35–38^. However, in these previous studies, the in vivo environment is modeled in vitro by confinement using solid flat surfaces. Whilst, the lumens within the fallopian tube are curved, with radii of curvature ranging from ~20 μm to over 150 μm (refs. ^3,5,18^), formed via soft epithelial surfaces. The mechanical properties of this epithelium (with elasticity and stiffness of 10–10^3^ Pa) is considerably different to the rigid flat surfaces in common laboratory dishes or microfabrication materials that are six orders of magnitude stiffer (e.g., polystyrene, polydimethylsiloxane or glass have elasticity and stiffness of 10^6^–10^9^ Pa)^39,40^.

Droplet microfluidics is well suited to the generation of soft curved interfaces of controlled shape and size^41^ for studying sperm behavior, providing an environment that closely mimics the architecture and mechanical properties of the female fallopian tube. In these systems, immiscible fluid phases form droplets of one phase within the other^42^. This compartmentalization in droplets has proved a breakthrough in the biomedical area for applications in high-throughput drug screening^42–44^, tissue and protein engineering^45,46^, and single cell analysis^47^. For example, droplets containing cells have previously been used to transport live organisms including yeast and zebrafish embryos^48,49^. Retention of zebrafish embryo viability for up to 2 hours during transportation shows the capability of droplet microfluidics to handle cells as large as 500 μm in diameter^48^. Individual live cells have also been isolated in droplets to study protein expression and antibody secretion in a well-controlled and monitored vessel^47,50^. Such single cell encapsulation of live cells in droplet microfluidics points to an opportunity to study sperm dynamics at curved liquid–liquid interfaces, representing a more physiologically relevant interface than solid flat surfaces.

Here, using droplet microfluidics (Fig. 1b), we observe and quantify sperm behavior at soft curved interfaces using individual sperm encapsulated in droplets (ranging in size from 30 µm to 140 µm). We find that surface curvature triggers a dynamic response mechanism in sperm to switch from a progressive surface-aligned motility mode at smaller curvatures (i.e., larger droplets >100 μm in radius) to an aggressive surface-attacking motility mode at bigger curvatures (i.e., smaller droplets <50 μm in radius). In the aggressive attacking mode, sperm swim at an acute angle (to the normal of the tangent) and are constantly in contact with the interface, spending 0.037 ± 0.007 s μm^−1^ at the surface–for a 1.66-fold longer period of time than a sperm in the progressive motility mode. Using a theoretical model, we reveal that hydrodynamic effects lead to an active response mechanism in sperm to decrease their flagellar wave amplitude by up to 66% at larger curvatures (smaller droplets). These findings highlight the role of changes in mammalian fallopian tube geometry^1,4^ to either guide the sperm towards the site of fertilization or facilitate sperm–egg interaction. Specifically, the lower curvature of the epithelial tissue in the isthmus encourages a surface aligned motility mode for sperm navigation, while higher curvatures in the ampulla activates an aggressive-surface attacking motility mode to encourage sperm–epithelial cell contact thus facilitating sperm capacitation and ultimately fertilization potential.

## Results

### Sperm motility modes at physiologically relevant curvatures

Individual bull sperm were encapsulated in droplets with radii ranging from 30 to 140 μm, and imaged for 12 s (Fig. 1b; Methods; see Supplementary Fig. 1 and Supplementary Table 1 for the size of analyzed droplets). This range of droplet size represents the complex epithelial tissue in the female fallopian tube where the curvature of folded lumens varies from 20 to over 150 μm in radius (Fig. 1a). In smaller droplets with <50 µm-radius but large enough to fit a sperm cell (≥36 μm in radius), sperm were observed to swim consistently in contact with and almost normal to the interface in an ‘aggressive attacking mode’ (Fig. 1c; Supplementary Movies 1 and 2). In this attacking mode, sperm always followed the droplet boundary to swim along a perfect circle that was only 0.6 ± 0.4 μm smaller in radius than the droplet radius (*n* = 45; Supplementary Figs. 2 and 3), by constantly pushing against the interface the instantaneous swimming trajectory was confined close to the average path (Fig. 1d; Methods). It is noteworthy that for droplets <36 μm in radius, as the droplet diameter was smaller than the cell length, sperm were observed to exhibit a compass-like behavior, in which the full body length was aligned normal to the interface and the head was pressing against it, rarely completing any revolution around the droplet (Supplementary Movie 3). These droplet sizes (<36 μm-radius) were not included in this study as in such small droplets, the full sperm length (~70 μm) was more than the diameter of droplets enclosing them (see Supplementary Fig. 1 and Supplementary Table 1 for the size of analyzed droplets). In larger droplets, ranging in radius from 50 to 100 μm, sperm were still swimming pushing against the interface, but were observed to intermittently depart from, or align parallel to, the interface, swimming in a ‘transition mode’ (Fig. 1e, f; Supplementary Movies 1 and 4). For droplets >100 μm in radius, in contrast to the attacking mode, sperm were observed to routinely align parallel to, or depart from, the interface (Fig. 1g; Supplementary Movies 1, 5 and 6) in a ‘progressive surface aligned motility mode’. In the progressive mode, sperm swam freely along a circular average path that was up to 6.8 ± 2.5 μm smaller in radius than the droplet (*n* = 45; Supplementary Figs. 2 and 3), with the instantaneous swimming trajectory oscillating around the average path (Fig. 1h). It is noteworthy that the change in the radius of droplet from 30 µm to 140 µm has a negligible effect on the Laplace pressure^51,52^ inside the droplet (less than 1% of the baseline atmospheric pressure, see Supplementary Note 1), without considerable effect on the observed swimming behavior. These results demonstrate that surface curvature alters sperm motion, guiding progressive motility at lower curvatures, and promoting increased and prolonged surface contact at higher curvatures.

**Fig. 2:**
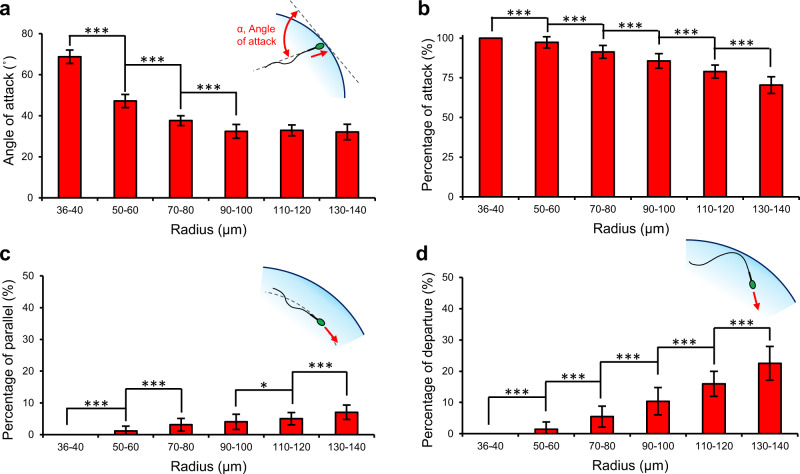
Characterization of sperm motility modes at curved interfaces. **a** Angle of attack, *α*, and the percentage of the 12 s tracked swimming trajectory in which the sperm is (**b**) attacking (2° < *α* < 90°), (**c**) swimming parallel to (−2° < *α* < 2°), and (**d**) departing (*α* < −2°) the interface in droplets ranging from 36 to 140 μm in radius. Values are reported as mean ± s.d. (*n* = 45, 46, 45, 46, 45, and 45 biologically independent cells examined over 5, 4, 4, 4, 5, and 4 independent experiments for sperm swimming in 36–40 µm, 50–60 µm, 70–80 µm, 90–100 µm, 110–120 µm and 130–140 µm radius droplets, respectively), *P* values were determined using one-way ANOVA, *P ≤ 0.05, **P ≤ 0.01, ***P ≤ 0.001 (for exact *P* values see Supplementary Table 3), and for data distribution see Supplementary Fig. 5. Source data are provided as a Source Data file.

We characterized the swimming behavior of sperm at curved interfaces (Fig. 2 and Supplementary Table 2) by tracking sperm for 12 s in droplets ranging in radius from 36 µm to 140 µm. With respect to the orientation of sperm at the interface, Fig. 2a shows the average angle between the longitudinal axis of sperm and the tangent line to the droplet, hereafter referred to as the angle of attack, *α* (see Supplementary Fig. 4). For droplets with radius smaller than 100 µm, the angle of attack exhibited a negative correlation with the droplet size. Specifically, the angle of attack decreased significantly by 54% from 69 ± 3° to 32 ± 3° by increasing the droplet radius from 36 µm to 100 µm, respectively, and then plateaued at 32 ± 4° for larger droplets. For a pusher microswimmer such as sperm, both the conical envelope of the flagellar wave^26,30^, and the hydrodynamic flow field around the tail^53,54^ interact with the interface to define the angle of attack. At curvatures smaller than 100 µm in radius, the interaction between sperm flagellum and the elevated surface upstream of the sperm head amplifies both the hydrodynamic effects and the steric repulsion to align the cell at an acute angle with respect to the interface (Supplementary Movies 1–5). However, a curvature larger than 100 µm in radius essentially acts as a flat surface to guide the sperm, with the conical envelope of the flagellar wave almost aligned with the surface, resulting in an angle of attack of ~30° (Supplementary Movies 1, 5 and 6).

In the attacking mode (specifically, for curvatures smaller than 50 µm in radius), sperm consistently (100% of the 12 s-tracked trajectory) aligned at an acute angle with the interface (29° < *α* < 90°) to attack and swim in contact with the interface (Fig. 2; also see Supplementary Fig. 5), with the angle of attack always greater than 61°. This attacking behavior in smaller droplets (36 μm to 40 μm in radius) was found to be independent of sperm capacitation status. Specifically, by repeating the experiment with a sample treated with heparin (see Methods), the percentage of capacitated sperm in the sample increased from 31 ± 4% to 82 ± 2% (fully and partially capacitated, *n* = 100, see Supplementary Fig. 6), but the angle of attack was almost constant (increasing from 69 ± 3° to 70 ± 2°, *n* = 45, *P* = 0.071, not statistically significant, see Supplementary Figs. 7a and 8) and cells were always observed to attack the interface (see Supplementary Fig. 7b). Notably, the range of curvature in the attacking mode is relevant to that of the later part of the female fallopian tube in ampulla, close to the site of fertilization, where physical contact between sperm and the epithelial surface is necessary for capacitation^8^. In the transition and progressive motility modes, the tendency of sperm to attack the interface decreased linearly (*R*^2^ = 0.98, *P* ≤ 0.001 with Pearson correlation) with increasing droplet size (Fig. 2b) from 97 ± 4% in 50 µm-radius droplets to 70 ± 5% in 140 µm-radius droplets. In contrast, the tendency of the sperm to swim parallel with (−2° < *α* < 2°) or depart from (*α* < −2°) the interface increased from 1 ± 1% and 1 ± 1% in 50 µm-radius droplets to 7 ± 2% and 22 ± 5% in 140 µm-radius droplets, respectively (Fig. 2c, d). The results indicate that sperm exhibit a greater tendency to attack and swim in contact with the surface at higher curvatures, whilst at lower curvatures the sperm navigate along the interface.

**Fig. 3:**
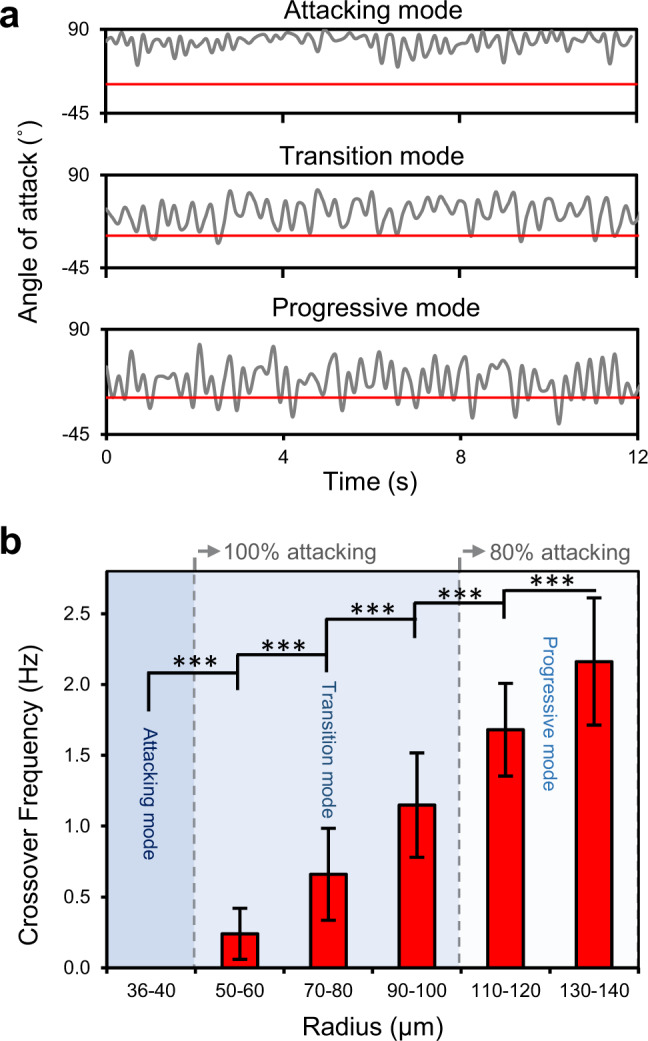
Characterization of the temporal behavior of sperm in the attacking, transition, and progressive modes. **a** Representative variations of the angle of attack along the 12 s tracked swimming trajectories for sperm swimming in the attacking, transition, and progressive modes. The horizontal red line indicates the threshold angle of attack of 2° for the sperm to align parallel to or depart from the interface. **b** Crossover frequency as a function of droplet radius. Values are reported as mean ± s.d. (*n* = 45, 46, 45, 46, 45, and 45 biologically independent cells examined over 5, 4, 4, 4, 5, and 4 independent experiments for sperm swimming in 36–40 µm, 50–60 µm, 70–80 µm, 90–100 µm, 110–120 µm, and 130–140 µm radius droplets, respectively), *P* values were determined using one-way ANOVA, ***P ≤ 0.001 (for exact *P* values see Supplementary Table 3), and for data distribution in (**b**) see Supplementary Fig. 10. Source data are provided as a Source Data file.

Figure 3 quantifies the temporal behavior of sperm to crossover from an attacking orientation to a parallel or departing orientation. The crossover frequency is the frequency at which the angle of attack crosses the threshold angle of 2° (Fig. 3a and Supplementary Fig. 9), switching from attacking mode to either transition or progressive mode. The crossover frequency was zero for sperm in the attacking mode, and increased significantly (*R*^*2*^ = 0.99, *P* ≤ 0.001 with Pearson correlation) in the transition and progressive modes, from 0.24 ± 0.18 Hz in 50 µm-radius droplets to 2.16 ± 0.45 Hz in 140 µm-radius droplets (Fig. 3b and Supplementary Table 2). Sperm exhibited sustained attacking behavior (radius of curvature <50 µm) to align at acute angle with the interface for the whole 12 s of the tracked trajectory (Fig. 3a; also see Supplementary Fig. 9), with small variation in the angle of attack and without any tendency to crossover to parallel or departing orientation (Fig. 3b; also see Supplementary Fig. 10). However, in the transition mode, sperm showed an intermittent behavior, switching from a sharp angle of attack to a parallel or departing orientation at irregular time intervals (happening once per every 1–4 s, depending on the radius of curvature). In the progressive mode, this behavior became more stable and sperm exhibited an alternating behavior to regularly crossover between attacking and non-attacking orientations every 679 ± 433 ms (*n* = 20), regularly detaching from the interface. Notably, the modulus of the angle of attack was always smaller than 90° in all the swimming modes, demonstrating that the motion around the interface is unidirectional, hence, the sperm is effectively guided along the interface.

### Kinematics of sperm motility at curvatures

**Fig. 4:**
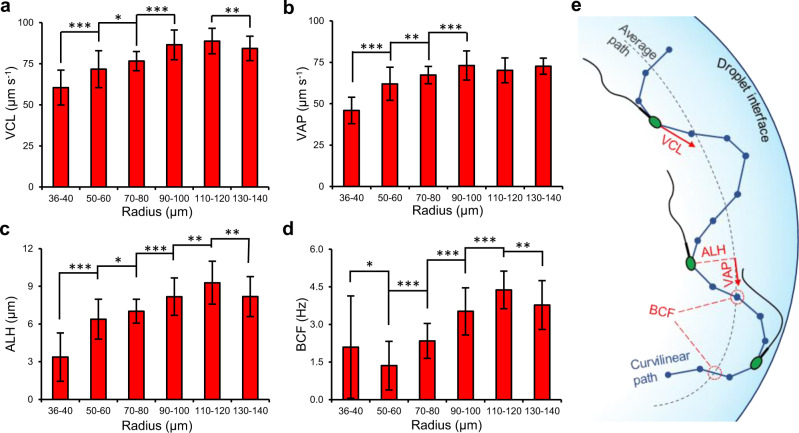
Sperm motility parameters at curved interfaces. **a** Curvilinear velocity (VCL), (**b**) average path velocity (VAP), (**c**) amplitude of lateral head displacement (ALH), and (**d**) beat cross frequency (BCF) for sperm swimming in droplets ranging in radius from 36 µm to 140 µm. Values are reported as mean ± s.d. (*n* = 45, 46, 45, 46, 45, and 45 biologically independent cells examined over 5, 4, 4, 4, 5, and 4 independent experiments for sperm swimming in 36–40 µm, 50–60 µm, 70–80 µm, 90–100 µm, 110–120 µm and 130–140 µm radius droplets, respectively), *P* values were determined using one-way ANOVA, *P ≤ 0.05, **P ≤ 0.01, ***P ≤ 0.001 (for exact *P* values see Supplementary Table 3), and for data distribution see Supplementary Fig. 11. **e** Schematic representation of the measured sperm motility parameters. Source data are provided as a Source Data file.

Figure 4 details the key motility parameters for sperm swimming in attacking, transition and progressive modes as a function of droplet radius (also see Supplementary Fig. 11 and Supplementary Table 2). As shown in Fig. 4e, curvilinear velocity (VCL) and average path velocity (VAP) are time-average velocities of the sperm head along its instantaneous and averaged trajectories, respectively. The measured VCL and VAP of sperm in the attacking mode (droplet radius < 50 µm) were significantly lower by at least 17% (from 60 ± 11 µm s^−1^ to 72 ± 11 µm s^−1^, Fig. 4a, *P* ≤ 0.001) and 26% (from 46 ± 8 µm s^−1^ to 62 ± 10 µm s^−1^, Fig. 4b, *P* ≤ 0.001), respectively, compared with sperm in the transition mode (in 50–110 µm droplets). VCL and VAP slightly increased in the transition mode for sperm in larger droplets, and plateaued at 87 µm s^−1^ and 72 µm s^−1^ for sperm in the progressive mode (droplet radius > 110 µm). The results demonstrate that sperm in the attacking mode are 33% slower than sperm in the progressive mode, prolonging the physical interaction between sperm head and the interface by 203% at tight curvatures. The amplitude of lateral head displacement (ALH; time-average deviation of the sperm head from its average path) and beat cross frequency (BCF; the frequency at which the instantaneous trajectory crosses the average path trajectory) are shown in Fig. 4c, d. Similar to VCL and VAP, ALH and BCF were also significantly lower, by 59% (decreasing from 8.2 ± 1.6 µm to 3.4 ± 1.9 µm, *P* ≤ 0.001) and 45% (decreasing from 3.8 ± 1.0 Hz to 2.1 ± 2.0 Hz, *P* ≤ 0.001) in the attacking mode compared to the progressive mode (Fig. 4c, d), demonstrating a highly restricted flagellar motion and a significant reduction in head oscillation in the attacking mode. These reductions in beating amplitude and frequency contribute to a reduction in the progressive velocity^55^. The increased proximity to the interface at tight curvatures (<50 µm in radius) dampens the flagellar motion, due to increased drag coefficients and friction^55,56^ to reduce the ALH and restrict the instantaneous swimming path close to the average path (Fig. 4e). Straight line velocity (VSL; the straight-line distance between the first and the last sperm tracking point divided by the total tracking time) and linearity (LIN; ratio of VSL to VCL) were almost constant for sperm swimming at curvatures <80 μm in radius, then increased between 80-μm-radius and 90 μm-radius curvatures (*P* ≤ 0.001) but plateaued thereafter (see Supplementary Figs. 12 and 13). For wobble (WOB; ratio of VAP to VCL) and straightness (STR; ratio of VSL to VAP), no clear trend was observed for sperm swimming at different curvatures (see Supplementary Figs. 12 and 13), as the sperm swim along a tight concentric circle inside the droplet dictated by the droplet radius (Fig. 1d, f, h), preventing the cell from swimming along a straight path.

## Discussion

We used droplet microfluidics to study sperm behavior at soft curved interfaces, closely mimicking the curvature^4^ and mechanical properties^39,40^ of folded epithelial tissue within the female fallopian tube. Our results reveal that hydrodynamic effects activate a response mechanism in sperm to switch from a ‘progressive motility’ mode at low curvatures (>100 µm in radius) to a distinct ‘aggressive surface-attacking motility’ mode at tight curvatures (<50 µm in radius). In the attacking mode, the sperm head is aligned at an acute angle and is consistently in contact with the interface, in strong contrast to the progressive mode observed at low curvatures and planar surfaces, where sperm routinely align parallel or depart from the interface. Sperm also exhibit a ‘transition motility mode’ at moderate curvatures (ranging in radius from 50 µm to 100 µm), with mixed characteristics of progressive and attacking modes. The average angle of attack for sperm in the attacking mode was found to be 69 ± 3°, which reduced significantly by 53% in the progressive mode and plateaued at ~30°. Several factors, associated with short-range steric interactions^22,53^ and long-range hydrodynamic effects^28,54^, contribute to orient the sperm at curvatures, including: (i) contact interactions between sperm tail and the elevated curved surface upstream of sperm (see Supplementary Movie 1–6), (ii) increased drag coefficients and friction experienced by the sperm due to nearby boundaries^55,56^ particularly upstream of the cell, and (iii) the interaction between the hydrodynamic flow field around the sperm tail and the elevated interface on the two sides of sperm^28,57^. Due to these interactions, in the aggressive attacking mode, sperm always (100% of the trajectories) swim at an acute attack angle, relatively normal to the interface. However, in the progressive mode, sperm exhibit a lower tendency to attack the interface (lower than 75% of the trajectories), rather they more routinely align parallel (6%) or depart (19%) the interface every 679 ± 433 ms.

To fully interpret the experimental results, we developed a simple theoretical model to estimate the angle of attack using similarity of triangles (see Supplementary Note 2). For a sperm of length *l *and beating amplitude of $\delta$ with the conical envelope of the flagellar wave aligned with a curvature of radius *R*, the angle of attack can be estimated as:

1 $$\alpha ={{\rm{sin }}}^{-1}\frac{\sqrt{{\delta }^{2}+{l}^{2}}}{2R}+{{\rm{tan }}}^{-1}\left(\frac{\delta }{l}\right)$$

From our experimental measurements for sperm freely swimming near a planar surface, the total length of bull sperm was ~70 µm, beating with a relatively fixed flagellar wave amplitude of ~15 µm at the end of the tail, in agreement with previous studies^58–60^.

**Fig. 5:**
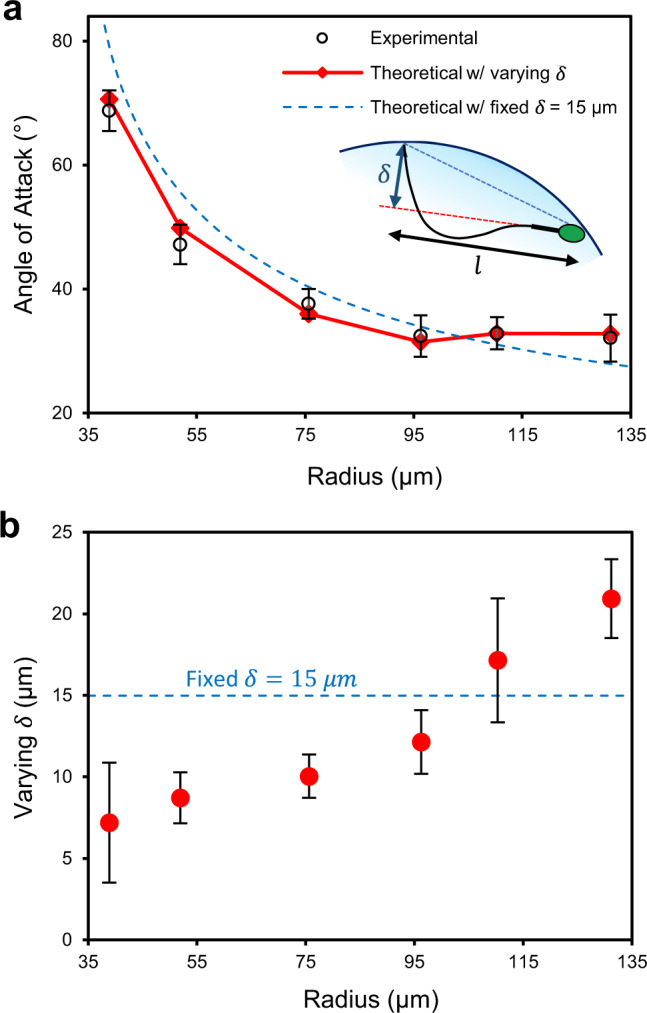
Theoretical model for estimating the angle of attack. **a** Angle of attack from experimental measurements as compared with theoretical values with fixed (dashed line) and varying (solid line) flagellar wave amplitude, $\delta$, as a function of droplet radius. Schematic representation of the reference system and modeled sperm shown by the inset. **b** Experimentally measured flagellar wave amplitudes as a function of droplet radius. Values are reported as mean ± s.d. (*n* = 45 cells examined over 4 independent experiments). Source data are provided as a Source Data file.

Figure 5 compares the experimentally measured angle of attacks with estimated values obtained from the theoretical model for experimentally measured beating amplitudes (i) near a planar surface (theoretical model with fixed $\delta$) and (ii) at curved interfaces (theoretical model with varying $\delta$). Using a fixed experimental $\delta$ of 15 µm in the theoretical model, the model deviates from experimental results, overestimating the angle of attack by 14% for 39 µm droplets and underestimating the angle of attack by 13% for 131 µm droplets, while following almost the same trend. We also experimentally measured the amplitude of flagellar beating for sperm in droplets of different curvatures (Fig. 5b). The results reveal that, instead of having a fixed beating amplitude of 15 μm, sperm decrease their flagellar wave amplitude by 66% at high curvatures (from 20.9 µm in 131 µm-radius droplets to 7.2 µm in 39 µm-radius droplets). The accuracy of the theoretical model to predict the angle of attack improved considerably when varying beating amplitude of sperm at different curvatures were used in the model (i.e., theoretical model with varying $\delta$ from experiments), predicting the angle of attack with less than 3% error and following the same trend. These findings suggest that sperm beating amplitude changes while adapting to varying environments with different radius of curvatures. The effect of high curvatures to reduce the flagellar wave amplitude, as also suggested by lower ALH values (Fig. 4c), contributes to reduce the swimming velocity by 33% in the attacking mode, and to prolong the physical interaction between the sperm head and the interface by up to 203%. This correlation highlights the potential importance of attacking mode for sperm in regions of the fallopian tube where physical interaction between sperm and the epithelial tissue is crucial for capacitation^8^.

The change in the flagellar beating amplitude observed here is potentially attributed to the interplay between the intercellular mechanisms of flagellar activity, the increased hydrodynamic drag and the additional spatial restrictions at tight curvatures. The flagellar waveform in sperm is produced due the structure of the axoneme at the central core of the flagellum^2^. The axoneme includes nine pairs of outer microtubule doublets that are connected to each other by nexin links and to a central pair of microtubules via radial spokes, forming a 9 + 2 axoneme structure^2,61^. The ATP-activated dynein motors sequentially slide each of the outer nine microtubules over the neighboring doublet, which bend the flagellum due to spatial restrictions imposed by the radial spokes and nexin links, forming the flagellar wave^2,61^. The flagellar waveform is defined by balancing the active sliding forces of the dynein motors with hydrodynamic forces and passive forces (due to the axoneme and accessory structures surrounding the axoneme)^55,62^. Therefore, any change in the hydrodynamic flow field or external forces experienced by the flagellum can potentially contribute to a change of the flagellar wave. Our results indicate that, in the attacking mode at tight curvatures, the increased hydrodynamic drag and additional spatial restrictions (steric interactions) create a force imbalance to regulate the flagellar wave and reduce the beating amplitude, potentially by activating an intercellular regulation mechanism. Such dynamic constrains of sperm head and tail have been shown to increase the intercellular sliding force of dynein arms^63,64^, and reduce the sliding amplitude due to the large drag experienced by the flagellum^65^. In response to change in the medium viscosity^66,67^, a signal coordinated through radial spokes has been suggested as an intercellular response mechanism for regulating dynein sliding forces and power production. Similarly, the increased hydrodynamic drag for sperm at tight curvatures may cause the same effect to activate this intercellular mechanism. Our results are also in agreement with the switch-inhibition mechanism that suggests force imbalance on the two sides of the flagellum is required to inhibit dynein arms on one side of the cell and drive the flagellar wave^68^. At tight curvatures with sperm oriented almost normal to the interface, the hydrodynamic forces and steric interactions on the two sides of sperm are balanced more effectively (as compared with sperm at bigger curvatures), mainly due to almost identical proximity of sperm to elevated interfaces on the two sides, and therefore, act to reduce the bend in flagellar wave based on the switch-inhibition mechanism. Taken together, the results indicate that increased drag or additional spatial restrictions at tight curvatures can potentially cause a force imbalance, influence the intercellular sliding force of dynein arms or activate the switch-inhibition mechanism to reduce the flagellar beating amplitude.

With respect to reproduction, our findings reveal the role of increasing geometrical complexity and confinement of the female fallopian tube in natural fertilization^3,4^. As sperm progress through the fallopian tube, from uterotubal junction (300 µm in radius) to isthmus and ampulla, the tract becomes highly folded and confined with epithelial lumens narrowing towards the site of fertilization (radius of curvatures decreasing from over 150 to ~20 µm-radius in size)^3,5,18^, exposing sperm to epithelial surfaces of higher curvatures. The isthmus, in the beginning part of the fallopian tube, includes a thick muscular layer^4^, resulting in a simple geometry with relatively large radius of curvatures in the folded epithelial tissue. These low curvatures encourage progressive and transition modes to enable faster locomotion towards the site of fertilization. However, the increased curvature of the epithelial lumens in ampulla and infundibulum, closer to the site of fertilization, encourage the aggressive attacking mode to increase the physical contact between sperm and the epithelial tissue and to prolong this interaction, increasing the chance of sperm survival^8–10^ and stimulating capacitation^8^.

**Fig. 6:**
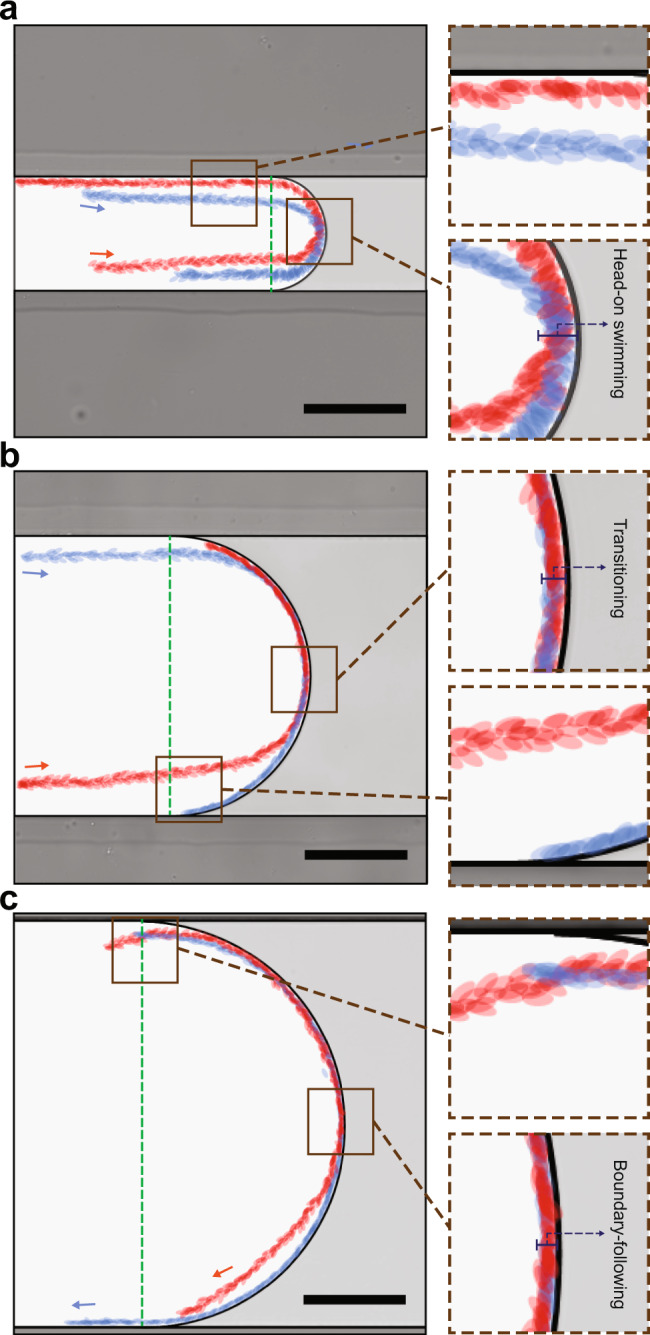
Sperm motility behavior at curved interfaces in microchannels that represent the increasing complexity of the female fallopian tube geometry. Overlaid trajectories of two representative sperm (head shown in red and blue) in microchannels that mimics the relevant range of curvatures in infundibulum, ampulla, and isthmus, with sperm exhibiting (**a**) head-on swimming behavior in the attacking mode, (**b**) a combination of head-on swimming and boundary following behavior in the transition mode, and (**c**) boundary-following behaviors in the progressive mode, respectively. Each experiment was repeated four times independently with similar results for (**a**–**c**). Images were contrast-adjusted for clarity. Scale bars, 100 µm.

To better highlight the role of surface curvature on regulating sperm behavior with respect to natural fertilization, when a straight swimmer faces a curvature, Fig. 6 shows overlaid trajectories of the same population of sperm in microchannels of different sizes, with curvatures that mimic relevant ranges of curvature in infundibulum, ampulla, and isthmus (see Supplementary Movie 7). In a 390 µm wide microchannel (radius of curvature of 195 µm), that is relevant to the geometry of the isthmus, sperm swim freely in the progressive mode along the interface by exhibiting the boundary-following behavior^23^, and a relatively fast locomotion (Fig. 6c), spending only 0.022 ± 0.001 s μm^−1^ at the curvature. The overlaid trajectory was confined within 5 ± 1 μm of the curvature, almost equal to the length of the minor axis of sperm head^60^, confirming a directed locomotion with a relatively parallel head orientation. In a smaller microchannel with 110 µm in width (radius of curvature of 55 µm), relevant to upstream regions of ampulla and infundibulum, sperm swim in the attacking mode by exhibiting a head-on interface swimming behavior (Fig. 6a), spending as high as 0.037 ± 0.007 s μm^−1^ at the curvature–for a 1.66-fold longer period of time than a sperm in the progressive mode. The thickness of overlaid sperm heads along the trajectory was 10 ± 1 μm, almost equal to the length of the major axis of sperm head^60^, confirming a head-on aggressive surface interaction.

Taken together, these findings show that the increasing geometrical complexity of the female fallopian tube alters sperm motion to guide the locomotion at lower curvatures but to increase and prolong surface contact at higher curvatures, enabling sperm capacitation and fertilization closer to the site of fertilization. These finding also provides additional insights into how surface curvature can be used to guide the locomotion for sperm selection applications or to increase surface interactions for understanding sperm attachment/detachment mechanisms. Motility of sperm at curvatures could also be relevant to the behavior of other pusher type microorganisms and eukaryotic microswimmers at curvatures, such as *E. col*i^28^, and motile *Caulobacter crescentus*^69^, particularly to understand their transport process in higher organisms.

## Methods

### Sperm sample preparation

Bull semen was purchased in straws form ABS Australia as commercially-available biological materials and stored in liquid nitrogen tank until used. The semen samples were collected as part of ABS Australia’s routine standard practice or provided to ABS Australia through a third-party company, and in accordance with the Animal Welfare Victoria requirements. Prior to the experiment, bull semen was thawed in 37 °C water bath for 5 min and removed from the straw using an artificial insemination syringe. The sample was then washed using a physiologically relevant HEPES-buffered salt solution (117 mM NaCl, 5.3 mM KCl, 2.3 mM CaCl_2_, 0.8 mM MgSO_4_, 1 mM NaH_2_PO_4_, 5.5 mM D-Glucose, 0.03 mM Phenol Red, 4 mM NaHCO_3_, 21 mM HEPES, 0.33 mM Na Pyruvate, 21.4 mM Na Lactate, supplemented with 1 mg ml^−1^ polyvinyl alcohol) via centrifugation at 200 g for 12 min, and then resuspended in 200 µl of pre-warmed buffer. Washed sperm sample were diluted to a concentration determined, by Poisson statistics^70^, to ensure the maximum probability of encapsulating single sperm in each droplet.

### Sperm capacitation assay

The Tyrode’s albumin lactate pyruvate (TALP) solution consisting of 100 mM NaCl, 3.1 mM KCl, 2 mM CaCl_2_, 0.4 mM MgCl_2_, 0.3 mM NaH_2_PO_4_, 25 mM NaHCO_3_, 10 mM HEPES, 1 mM Na Pyruvate, 21.6 mM Na Lactate, and 6 mg ml^−1^ BSA (Cohn’s fraction V, Sigma-Aldrich), supplemented with 10 μg ml^−1^ heparin sodium salt (Sigma-Aldrich) was used as the capacitation medium^71^. To prepare the sperm-TALP suspension, bull semen was washed using the TALP solution via centrifugation at 200 g for 12 min, and then resuspended in 200 µl of pre-warmed TALP solution (supplemented with 10 μg ml^−1^ heparin). The sperm-TALP suspension was then incubated at 38.5 °C in a cell incubator with 5% CO_2_ for 4 h, and suspension was gently mixed every half an hour^71^.

Sperm capacitation status was evaluated by monitoring the level of tyrosine phosphorylation^72,73^. Briefly, sperm-TALP suspension was diluted to a concentration of 4 × 10^6^ sperm per ml, and centrifuged at 600 g for 5 min. After removing 150 μl of the supernatant, the sample was mixed thoroughly with 50 μl of 4% paraformaldehyde in Phosphate-buffered saline (PBS) and left at room temperature for 10 min. The sample was then washed three times in PBS and centrifugation at 1200 g for 5 min before being resuspended in 200 µl. 30 µl of the sample was pipetted on a microscope slide and air dried overnight. The slide was then immunolabelled for phosphorylated tyrosine residues using 2 μg ml^−1^ anti-phosphotyrosine antibody, clone 4G10 (05–321, Sigma-Aldrich) as a primary antibody^72^. Sperm nuclei were stained with DAPI (1:500 dilution). Images of sperm were captured using an inverted fluorescence microscope (Olympus IX83, Japan) and at least 100 sperm were scored based on sperm tail staining status as fully capacitated (the full length of the tail labeled), partially capacitated (part of the sperm tail labeled), and non-capacitated (no labeling of the tail, see Supplementary Fig. 14).

### Device fabrication

A mask for a polydimethylsiloxane (PDMS) microfluidic device master mold was designed in AutoCAD software. The device comprises two inlets, one outlet, a 100 µm flow focusing junction for droplet generation and an expansion chamber (Fig. 1b; Supplementary Fig. 15). The expansion chamber was designed to reduce the progression speed of the droplets and so holding them in place for long-term imaging. The master mold was fabricated by patterning Hexamethyldisilazaneand AZ^®^ nLOF 2035 negative photoresist on a silicon wafer and then selectively etching the silicon using Oxford Plasma Lab100 System to achieve a desired depth of 62 µm. The top layer of the device was fabricated in PDMS (Silgards 184: Dow Corning, MI, USA) with 10:1 mixing ratio, and cured for 4 h at 70 °C on a hotplate. The inlet and outlet ports were then punched in the cured PDMS. Finally, the PDMS layer was bonded to a glass slide using an oxygen plasma. To study the interaction of the same population of sperm with interfaces of different curvatures a serpentine geometry with different microchannel width after each turn was used (Supplementary Fig. 16).

### Experimental procedure

A standard droplet microfluidic generation technique (flow focusing junction) was used, in which a constant flow of buffer and sample meet at a microfluidic junction resulting in monodisperse droplets encapsulating single sperm^74^. A synthetic and biocompatible oil (3M^TM^ Novec^TM^) was used as the continuous phase in which 2% of a biocompatible surfactant (Pico-Surf^TM^ 1, Sphere Fluidics, UK) was added to avoid droplets from merging with each other. A pressure pump (MFCS™-EZ, Fluigent system) controlled by MAESFLO™ 3.3.1 software was used to adjust the inlet pressures for both the dispersed (sperm & buffer) and continuous phases (oil and surfactant). After producing the droplets, the outlet pressure was adjusted so that the droplets stayed static in the expansion chamber where they were imaged. Between experiments, the ratio of the two inlet pressures was adjusted such that droplets of different sizes were generated. It is noteworthy that, due to the 62 μm height of the channel and relatively large size of the droplets, each droplet is essentially a flattened disk rather than a sphere and sperm motion is mainly limited into a two-dimensional (2D) plane, allowing for straight-forward visualization in 2D.

### Microscopy

An inverted fluorescence microscope (Olympus IX83, Japan) equipped with an ORCA-Flash4.0 V3 Digital CMOS camera (Hamamatsu Photonics, Japan) and a stage incubator was used to capture 20× and 60× magnification images of sperm in bright-field imaging modes at 15 and 25 frames per second using Olympus cellSens Dimension 2.1 software.

### Image analysis

The image processing software ImageJ was used to measure size of the droplets, process images, and manually track sperm, measure the flagellar wave amplitude, *δ*, and angle of attack, *α* (see Supplementary Fig. 4). The location of the sperm head was tracked manually in each droplet for 12 s (45 sperm were tracked in each case). A custom-written script in Matlab was then used to analyze the motility parameters for the tracked sperm and reconstruct the swimming trajectory. The motility parameters were defined according to World Health Organization (WHO) guidelines^75^ as: (i) curvilinear velocity (VCL): time-average velocity of the sperm head along its instantaneous trajectory; (ii) average path velocity (VAP): time average velocity of the sperm following its average trajectory; (iii) straight line velocity (VSL): the distance between the first and the last sperm tracking point divided by the total duration of the track segment; (iv) amplitude of lateral head displacement (ALH): time-average deviation of the sperm head from its average path; (v) beat cross frequency (BCF): the frequency at which the instantaneous sperm trajectory crosses the average path trajectory; (vi) linearity (LIN): ratio of VSL to VCL; (vii) wobble (WOB): the ratio of VAP to VCL; and (viii) Straightness (STR): the ratio of VSL to VAP. The angle of attack, *α*, was also measured manually as the angle between sperm axis and the tangent line to the droplet (see Supplementary Fig. 4). Crossover frequency was measured as the frequency at which the angle of attack crosses the threshold angle of 2°, demonstrating the number of switches from attacking mode to either transition or progressive mode per unit time. A statistical analysis was performed using one-way ANOVA between each two groups (see Supplementary Table 3 for full statistical analysis), where *P* less than 0.05 was considered significant (*P ≤ 0.05, **P ≤ 0.01, ***P ≤ 0.001).

### Reporting summary

Further information on research design is available in the Nature Research Reporting Summary linked to this article.

## Supplementary information

Supplementary Information

Description of Additional Supplementary Files

Supplementary Movie 1

Supplementary Movie 2

Supplementary Movie 3

Supplementary Movie 4

Supplementary Movie 5

Supplementary Movie 6

Supplementary Movie 7

Reporting Summary

## Data Availability

All microscopy images and relevant data supporting the findings of this study are available within the article and its Supplementary Information files or upon request from the corresponding author, Reza Nosrati, at reza.nosrati@monash.edu. A reporting summary for this Article is available as a Supplementary Information file. The female fallopian tube cross-section image in Fig. 1a was adopted without change from https://www.flickr.com/photos/euthman/2760474960/ (entitled “Normal Fallopian Tube, Human”, by Ed Uthman) under CC BY 2.0. Source data are provided with this paper.

## Source data

Source Data

## Code availability

Matlab version 9.7.0.1190202 (R2019b) was used to generate custom scripts to analyze the motility parameters for the tracked sperm and reconstruct the swimming trajectory. Please contact Reza Nosrati, at reza.nosrati@monash.edu, for access.
