# Supplementary material for: Curvature in the reproductive tract alters sperm–surface interactions
Source: Nat Commun. 2021 Jun 8;12:3446. doi: 10.1038/s41467-021-23773-x (PMC8187733; doi:10.1038/s41467-021-23773-x)
Supplement: Supplementary file 1 — Supplementary Information [file 41467_2021_23773_MOESM1_ESM.pdf]

Supplementary Information

Curvature in the reproductive tract alters sperm-surface interactions

Mohammad Reza Raveshi[1], Melati S. Abdul Halim[1], Sagar N. Agnihotri[1,2], Moira K. O'Bryan[3,4], Adrian Neild[1], and Reza Nosrati[1]

[1] *Department of Mechanical and Aerospace Engineering, Monash University, Clayton, Victoria 3800, Australia*
[2] *IITB-Monash Research Academy, IIT Bombay, Mumbai 400076, India*
[3] *School of Biological Sciences, Monash University, Clayton, Victoria 3800, Australia*
[4] *School of BioSciences, Faculty of Science, University of Melbourne, Parkville, Victoria 3010. Australia*

Correspondence should be addressed to A.N. (email: Adrian.Neild@monash.edu) and R.N. (email: Reza.Nosrati@monash.edu).

Supplementary Figures

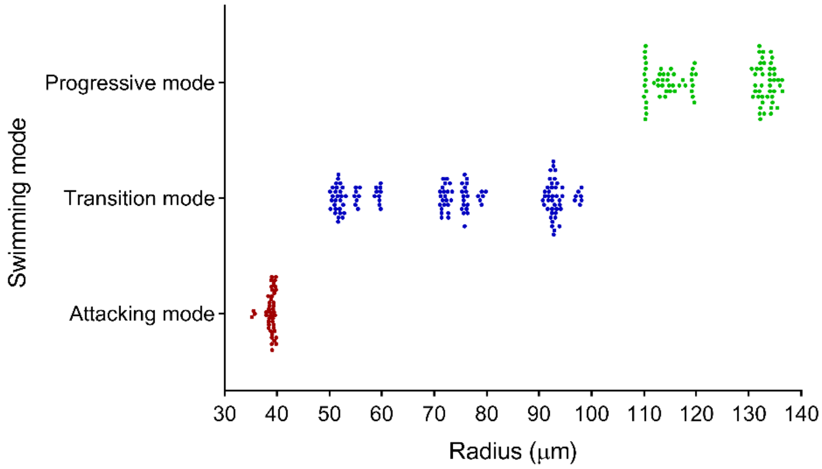

Supplementary Figure 1 | Distribution of droplet sizes analyzed under attacking, transition, and progressive motility modes. Red, blue and green symbols were used to indicate droplets analysed in the attacking, transition and progressive modes, respectively. Source data are provided as a Source Data file.

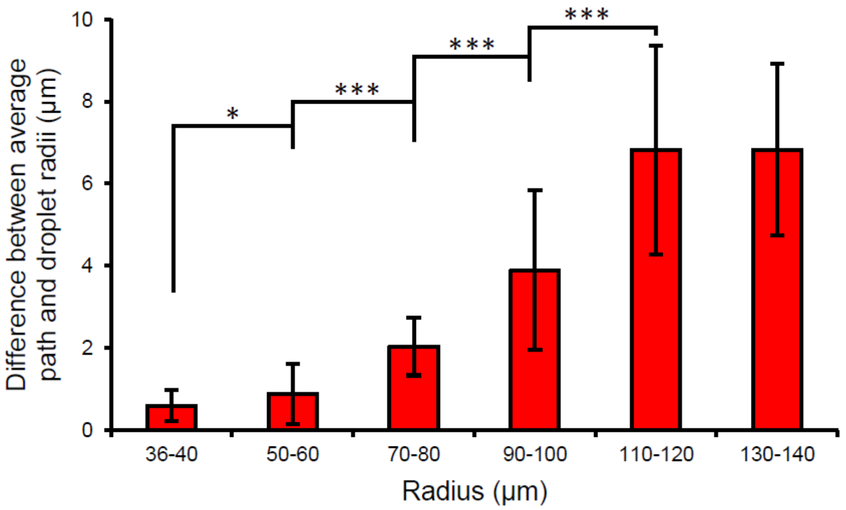

Supplementary Figure 2 | Difference between average path and droplet radius for three different modes of sperm swimming in droplets with different size. The distance between the boundary of the surface and the circular average path of the sperm during the different swimming modes increases while the droplet size increases. Values are reported as mean ± s.d. (*n*=45, 46, 45, 46, 45 and 45 biologically independent cells examined over 5, 4, 4, 4, 5 and 4 independent experiments for sperm swimming in 36-40 µm, 50-60 µm, 70-80 µm, 90-100 µm, 110-120 µm and 130-140 µm radius droplets, respectively), *P* values were determined using one-way ANOVA, $*P \leq 0.05$, $***P \leq 0.001$ (*P* values between adjacent columns are 2×10^{-2}, 2×10^{-11}, 4×10^{-8}, 2×10^{-8} and 0.99), for data distribution see Supplementary Fig. 3. Source data are provided as a Source Data file.

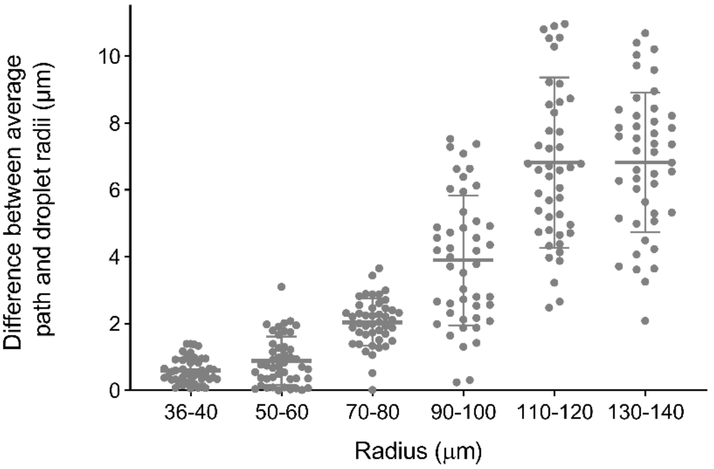

Supplementary Figure 3 | Swarm plot of difference between average path and droplet radius as a function of droplet radius, representing the underlying distribution of data points. Values are reported as mean ± s.d. (*n*=45, 46, 45, 46, 45 and 45 biologically independent cells examined over 5, 4, 4, 4, 5 and 4 independent experiments for sperm swimming in 36-40 µm, 50-60 µm, 70-80 µm, 90-100 µm, 110-120 µm and 130-140 µm radius droplets, respectively). Source data are provided as a Source Data file.

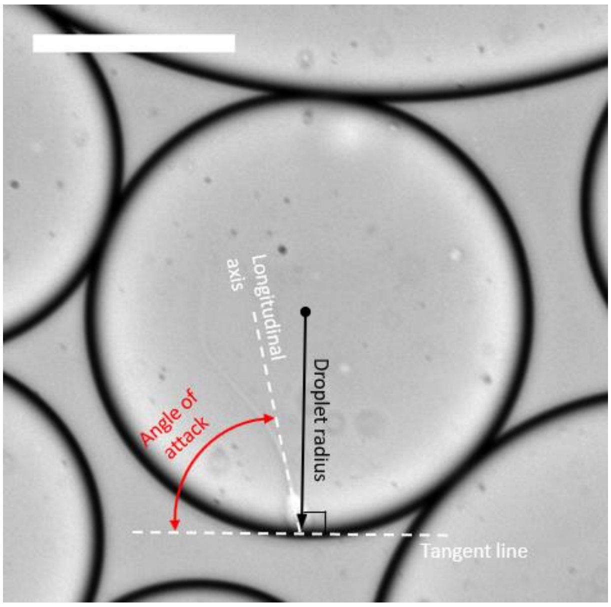

Supplementary Figure 4 | A representative image showing the method applied to manually measure the angle of attack. The angle between the longitudinal axis of sperm head and the tangent line to the droplet (normal to the intersecting droplet radius) was measured as the angle of attack. Each experiment was repeated 4 times independently with similar results. Scale bars, 50 µm.

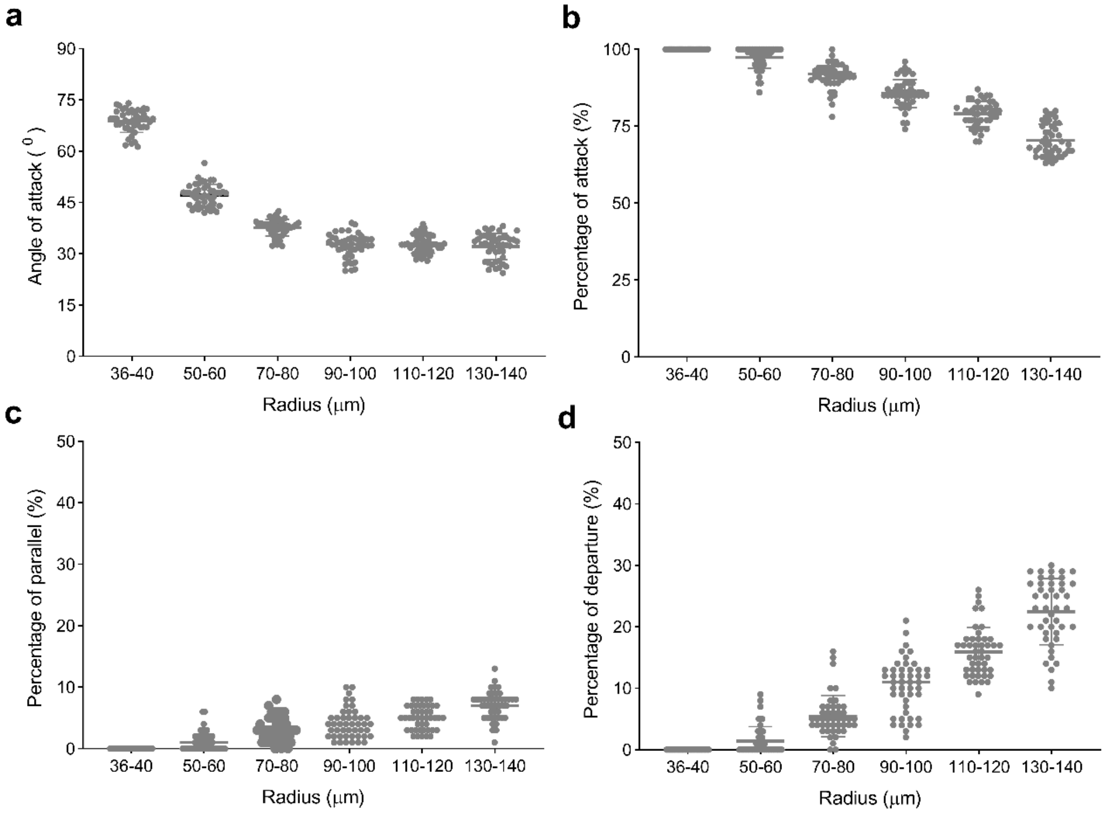

Supplementary Figure 5 | Swarm plots of sperm motility modes at curved interfaces representing the underlying distribution of data points. (**a**) Angle of attack, α, and the percentage of the 12 s tracked swimming trajectory in which the sperm is (**b**) attacking (2°<α<90°), (**c**) swimming parallel to (-2°<α<2°), and (**d**) departing (α<-2°) the interface in droplets ranging from 36 to 140 µm in radius. Values are reported as mean ± s.d. (n=45, 46, 45, 46, 45 and 45 biologically independent cells examined over 5, 4, 4, 4, 5 and 4 independent experiments for sperm swimming in 36-40 µm, 50-60 µm, 70-80 µm, 90-100 µm, 110-120 µm and 130-140 µm radius droplets, respectively). Source data are provided as a Source Data file.

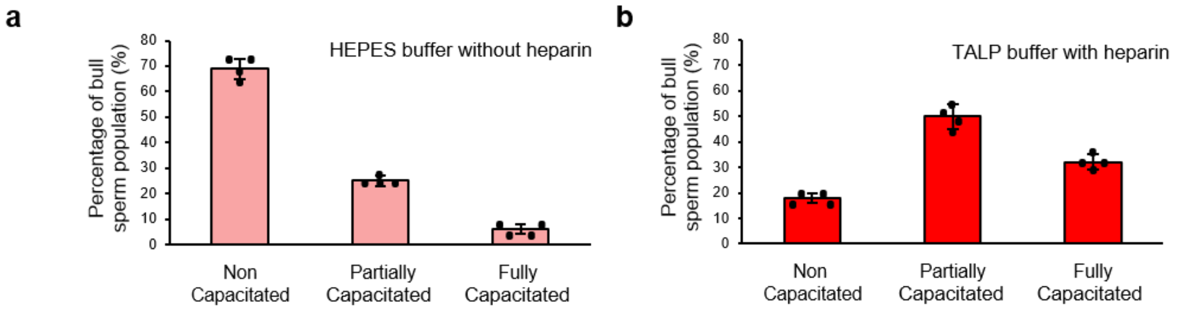

Supplementary Figure 6 | Sperm capacitation status. Percentage of fully capacitated, partially capacitated, and non-capacitated sperm in (**a**) HEPES buffer without heparin and (**b**) TALP buffer supplemented with 10 µg ml^{-1} heparin. Values are reported as mean ± s.d. (*n*=100 cells examined over 4 independent experiments), *P* values were determined using one-way ANOVA, (*P* values for non-capacitated, partially capacitated and fully capacitated sperm between **a** and **b** are 5×10^{-7}, 1×10^{-4} and 1×10^{-5}, respectively). Source data are provided as a Source Data file.

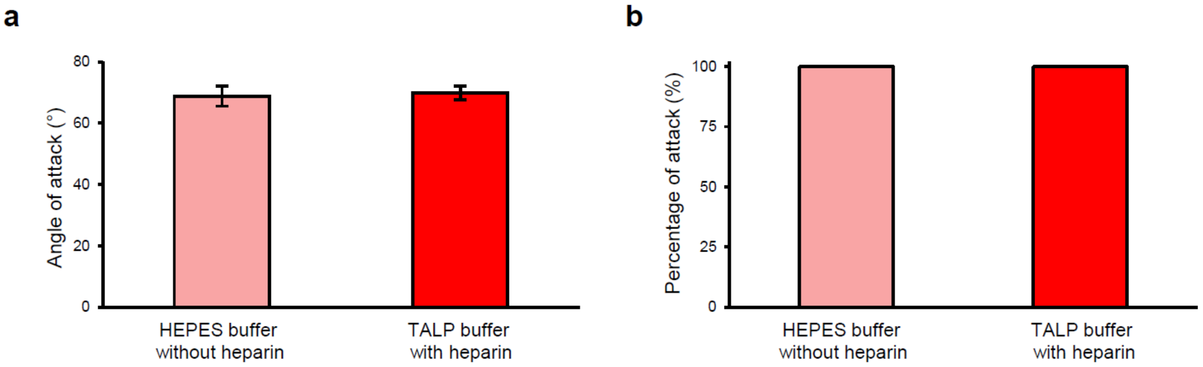

Supplementary Figure 7 | Characterization of sperm attacking motility mode (<40 µm-radius droplets) in HEPES buffer without heparin and TALP buffer with heparin. (**a**) Angle of attack, *α*, and (**b**) the percentage of the 12 s tracked swimming trajectory in which the sperm is attacking (2°<*α*<90°) the interface. Values are reported as mean ± s.d. (*n*=45 cells examined over 5 independent experiments), *P* values were determined using one-way ANOVA, *P*=0.071 in **a** and *P*=1 in **b**, and for data distribution in **a** see Supplementary Fig. 8. Source data are provided as a Source Data file.

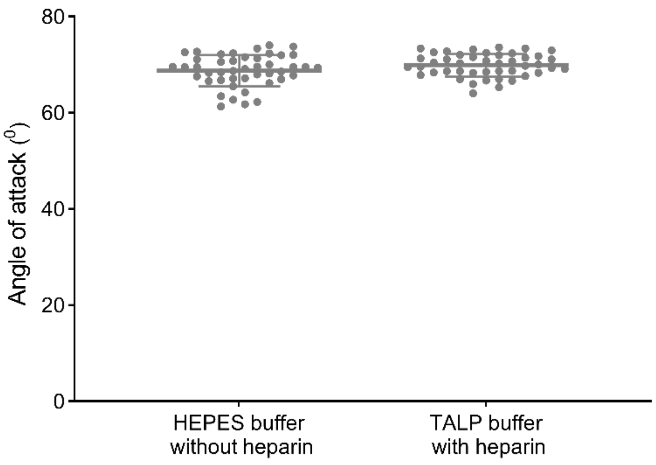

Supplementary Figure 8 | Swarm plot of angle of attack, α, in the attacking mode (<40 µm-radius droplets) in HEPES buffer without heparin and TALP buffer with heparin. Values are reported as mean ± s.d. (*n*=45 cells examined over 5 independent experiments). Source data are provided as a Source Data file.

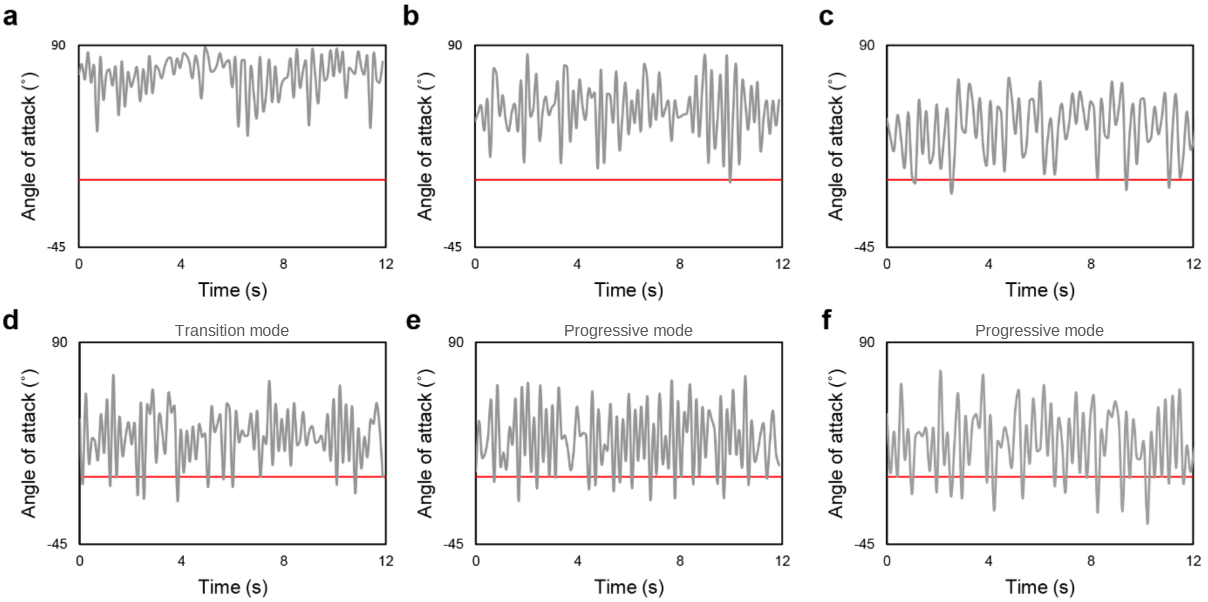

Supplementary Figure 9 | Temporal variations in the angle of attack for sperm in the attacking, transition, and progressive modes. Representative variations of the angle of attack along the 12 s tracked swimming trajectories for sperm swimming in droplets with radius raining from (**a**) 36 µm to 40 µm, (**b**) 50 µm to 60 µm, (**c**) 70 µm to 80 µm, (**d**) 90 µm to 100 µm, (**e**) 110 µm to 120 µm, and (**f**) 130 µm to 140 µm. Sperm are swimming in the attacking mode in **a**, in the transition mode in **b-d** and in the progressive mode in **e** and **f**. Source data are provided as a Source Data file.

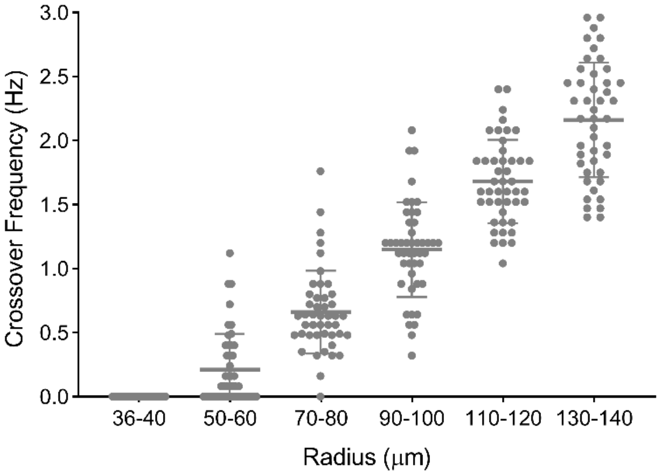

Supplementary Figure 10 | Swarm plot of crossover frequency as a function of droplet radius representing the underlying distribution of data points. Values are reported as mean ± s.d. (*n*=45, 46, 45, 46, 45 and 45 biologically independent cells examined over 5, 4, 4, 4, 5 and 4 independent experiments for sperm swimming in droplets ranging in radius from 36 µm-40 µm, 50 µm-60 µm, 70 µm-80 µm, 90 µm-100 µm, 110 µm-120 µm and 130 µm-140 µm, respectively). Source data are provided as a Source Data file.

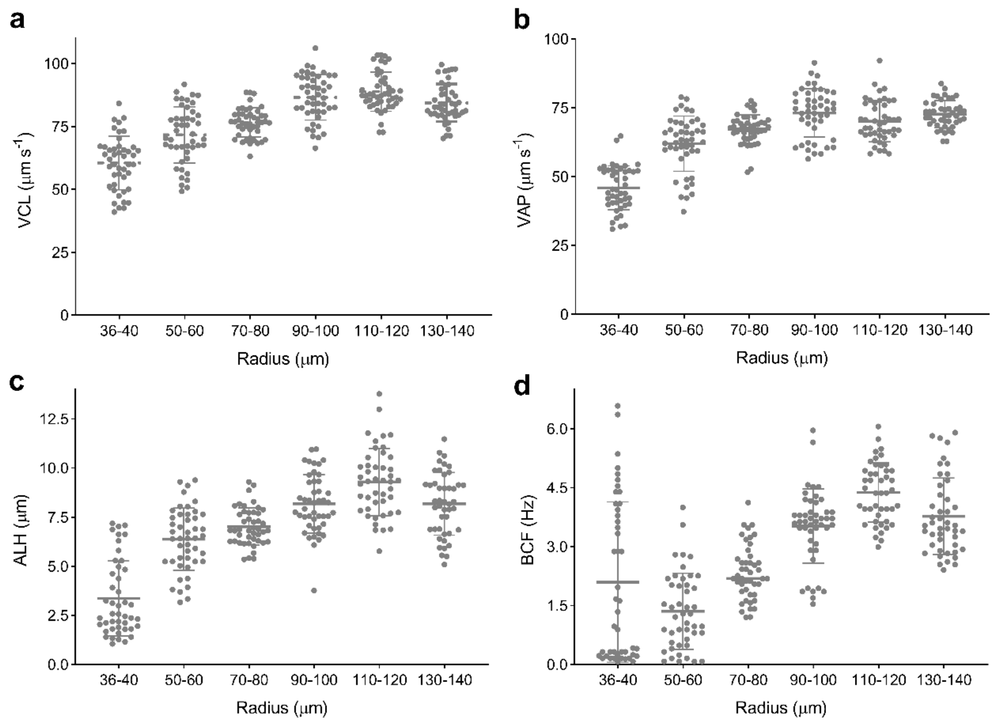

Supplementary Figure 11 | Swarm plots of sperm motility parameters at curved interfaces representing the underlying distribution of data points. (**a**) Curvilinear velocity (VCL), (**b**) average path velocity (VAP), (**c**) amplitude of lateral head displacement (ALH), and (**d**) beat cross frequency (BCF) for sperm swimming in droplets ranging in radius from 36 µm to 140 µm. Values are reported as mean ± s.d. (*n*=45, 46, 45, 46, 45 and 45 biologically independent cells examined over 5, 4, 4, 4, 5 and 4 independent experiments for sperm swimming in 36-40 µm, 50-60 µm, 70-80 µm, 90-100 µm, 110-120 µm and 130-140 µm radius droplets, respectively). Source data are provided as a Source Data file.

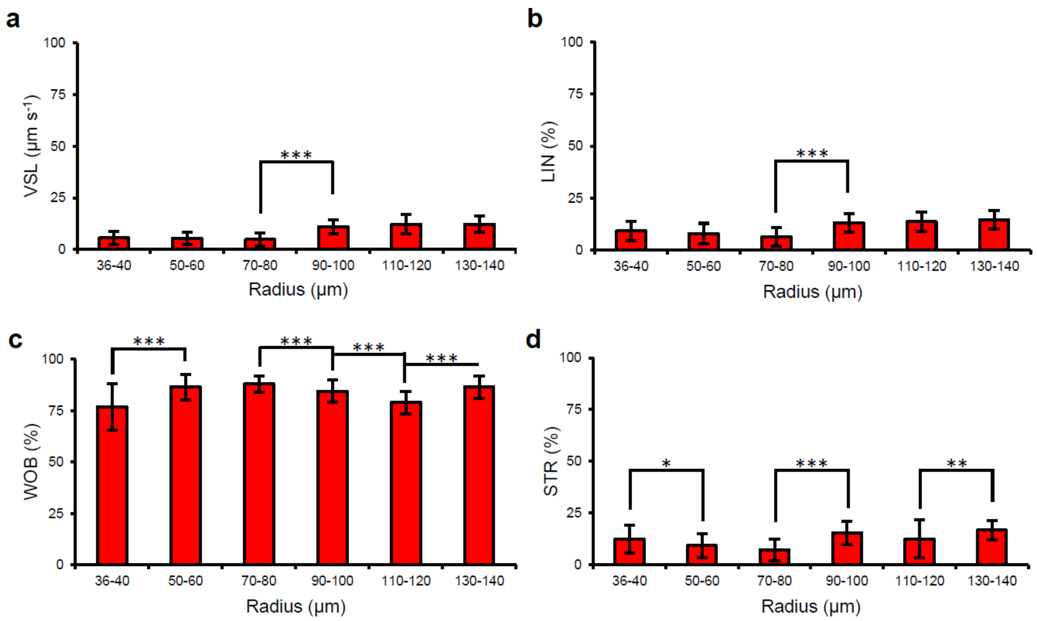

Supplementary Figure 12 | Sperm motility parameters at curved interfaces. (**a**) Straight line velocity (VSL), (**b**) linearity (LIN), (**c**) wobble (WOB), and (**d**) straightness (STR) for sperm swimming in droplets ranging in radius from 36 µm to 140 µm. Values are reported as mean ± s.d. (*n*=45, 46, 45, 46, 45 and 45 biologically independent cells examined over 5, 4, 4, 4, 5 and 4 independent experiments for sperm swimming in 36-40 µm, 50-60 µm, 70-80 µm, 90-100 µm, 110-120 µm and 130-140 µm radius droplets, respectively), *P* values were determined using one-way ANOVA, $^*P \leq 0.05$, $^{**}P \leq 0.01$, $^{***}P \leq 0.001$ (*P* values between adjacent columns are 0.73, 0.4, 1×10^{-13}, 0.12 and 0.97 in **a**, *P* values between adjacent columns are 0.26, 0.09, 2×10^{-10}, 0.53 and 0.35 in **b**, *P* values between adjacent columns are 2×10^{-6}, 0.22, 9×10^{-4}, 5×10^{-6} and 5×10^{-9} in **c** and *P* values between adjacent columns are 0.02, 0.08, 9×10^{-11}, 0.06 and 7×10^{-3} in **d**), and for data distribution see Supplementary Fig. 13. Source data are provided as a Source Data file.

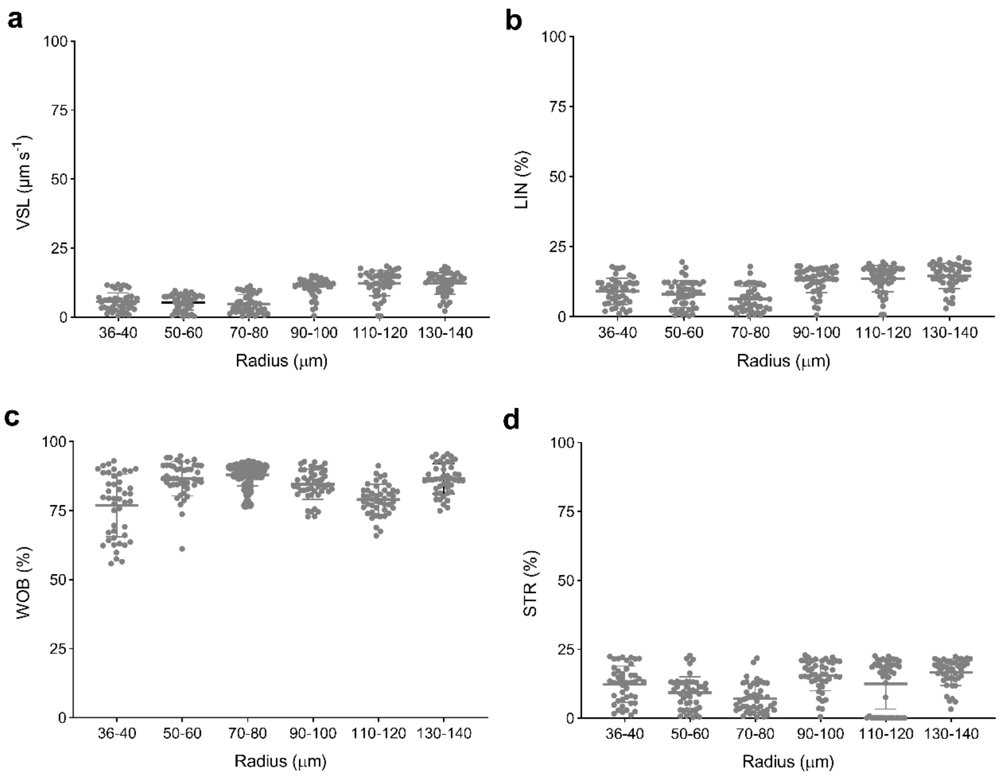

Supplementary Figure 13 | Swarm plots of sperm motility parameters at curved interfaces representing the underlying distribution of data points. (**a**) Straight line velocity (VSL), (**b**) linearity (LIN), (**c**) wobble (WOB), and (**d**) straightness (STR) for sperm swimming in droplets ranging in radius from 36 µm to 140 µm. Values are reported as mean ± s.d. (*n*=45, 46, 45, 46, 45 and 45 biologically independent cells examined over 5, 4, 4, 4, 5 and 4 independent experiments for sperm swimming in 36-40 µm, 50-60 µm, 70-80 µm, 90-100 µm, 110-120 µm and 130-140 µm radius droplets, respectively). Source data are provided as a Source Data file.

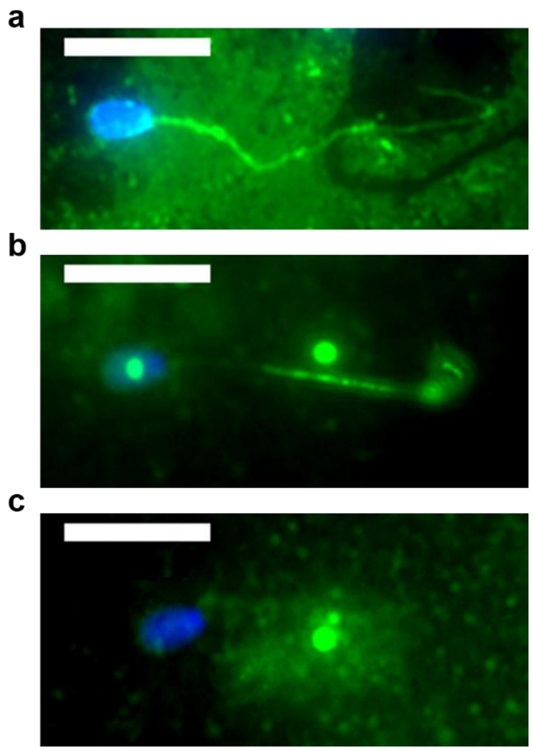

Supplementary Figure 14 | Sperm capacitation assay based on tyrosine phosphorylation. A representative fluorescent image of a (**a**) fully capacitated sperm with the full length of the tail labelled, (**b**) partially capacitated sperm with part of the sperm tail labelled, and (**c**) non-capacitated sperm with no-labelling of the tail. Each experiment was repeated 5 times independently with similar results for **a**-**c**. Scale bars, 25 µm.

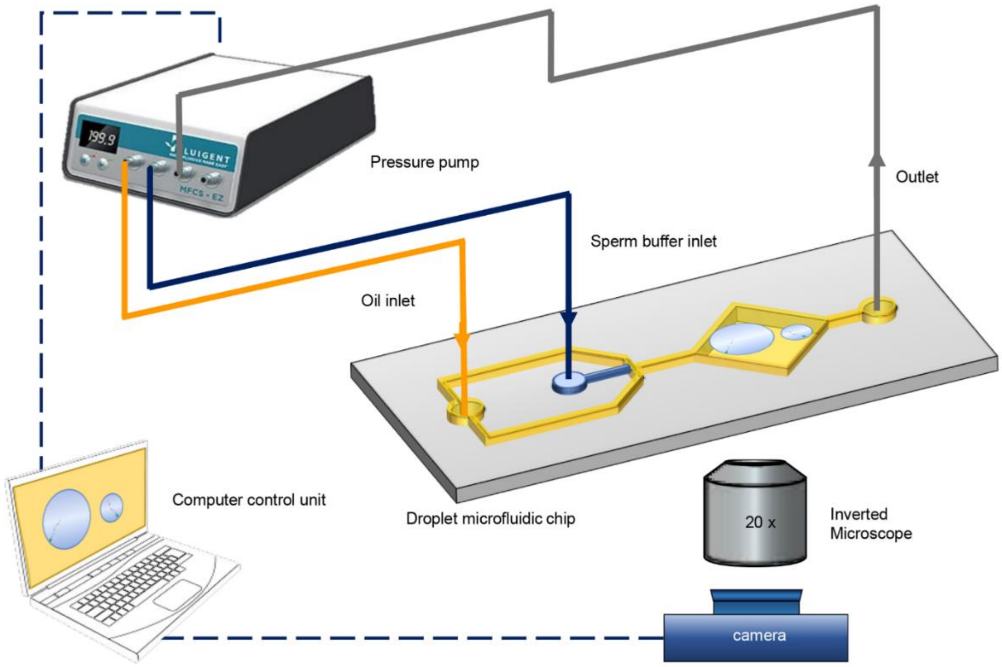

Supplementary Figure 15 | Schematic shows the setup used for droplet microfluidic based sperm motility analysing. The inlets and outlet of the droplet microfluidic system is connected to a microfluidic flow control system (MFCS^{TM}-EZ, Fluigent system). Setup includes ORCA-Flash4.0 V3 Digital CMOS camera (Hamamatsu Photonics, Japan), inverted fluorescence microscope (Olympus IX83, Japan) and Fluigent systems, connected to a computer for data collection.

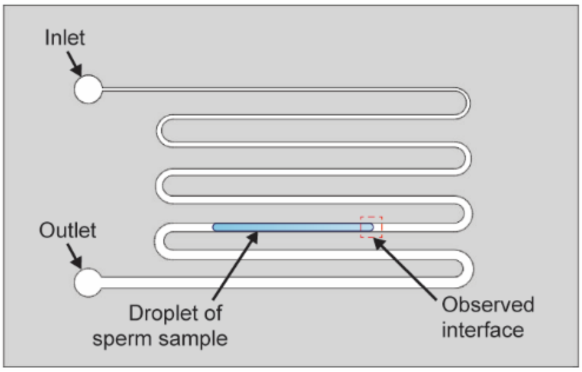

Supplementary Figure 16 | A microfluidic device with a serpentine geometry that includes microchannels of different width. The same droplet of sperm sample was moved in the device to study the interaction of the same population of sperm with interfaces of different curvatures.

Supplementary Tables

Supplementary Table 1 | Mean radius of droplets analyzed for each radius range. Values are reported as mean ± s.d.

Radius range (μm)	Number of analyzed droplets	Mean radius (μm)
36 - 40	45	38.8 ± 1.0
50 - 60	46	53.9 ± 3.3
70 - 80	45	74.4 ± 2.6
90 - 100	46	93.4 ± 2.0
110 - 120	45	114.2 ± 3.4
130 - 140	45	133.3 ± 1.5

Supplementary Table 2 | Sperm motility parameters at curved interfaces. Values are reported as mean ± s.d. (n≥45).

Parameter	Droplet radius (μm)					
	**36-40** (n=45)	**50-60** (n=46)	**70-80** (n=45)	**90-100** (n=46)	**110-120** (n=45)	**130-140** (n=45)
VCL (μm s^{-1})	60±11	72±11	77±6	87±9	89±8	84±7
VAP (μm s^{-1})	46±8	62±10	67±5	73±9	70±7	73±5
ALH (μm)	3.4±1.9	6.4±1.6	7.0±1.0	8.2±1.5	9.3±1.7	8.2±1.6
BCF (Hz)	2.1±2.0	1.4±1.0	2.3±0.7	3.5±0.9	4.4±0.8	3.8±1.0
Angle of attack (°)	69±3	47±3	38±2	32±3	33±3	32±4
Percentage of attack (%)	100±0	97±4	91±4	86±5	79±4	70±5
Percentage of parallel (%)	0±0	1±1	3±2	4±2	5±2	7±2
Percentage of departure (%)	0±0	1±1	5±3	10±4	16±4	22±5
Crossover frequency (Hz)	0.00±0.00	0.24±0.18	0.66±0.32	1.15±0.37	1.68±0.33	2.16±0.45

Supplementary Table 3 | Statistical analysis of sperm motility parameters. P values were calculated using one-way ANOVA based on at least 45 points for each range of radius of droplets, *P≤0.05, **P≤0.01, ***P≤0.001 and NS denotes not significant.

Group	a	b	c	d	e	f
Radius of droplet (μm)	36-40	50-60	70-80	90-100	110-120	130-140
n	45	46	45	46	45	45

Parameter/ Compared groups	a,b	a,c	a,d	a,e	a,f	b,c	b,d	b,e	b,f	c,d	c,e	c,f	d,e	d,f	e,f
VCL (μm s^{-1})	4×10^{-6} ***	5×10^{-14} ***	1×10^{-21} ***	6×10^{-25} ***	7×10^{-21} ***	2×10^{-2} *	4×10^{-10} ***	8×10^{-13} ***	2×10^{-8} ***	1×10^{-8} ***	6×10^{-13} ***	4×10^{-7} ***	2×10^{-1} NS	2×10^{-1} NS	7×10^{-3} **
VAP (μm s^{-1})	6×10^{-13} ***	5×10^{-26} ***	6×10^{-27} ***	1×10^{-25} ***	4×10^{-33} ***	4×10^{-3} **	2×10^{-7} ***	6×10^{-5} ***	1×10^{-8} ***	2×10^{-4} ***	4×10^{-2} *	3×10^{-6} ***	8×10^{-2} NS	8×10^{-1} NS	6×10^{-2} NS
ALH (μm)	2×10^{-12} ***	6×10^{-19} ***	5×10^{-23} ***	8×10^{-27} ***	4×10^{-22} ***	4×10^{-2} *	2×10^{-7} ***	1×10^{-12} ***	1×10^{-6} ***	3×10^{-5} ***	1×10^{-11} ***	6×10^{-5} ***	1×10^{-2} **	9×10^{-1} NS	2×10^{-3} **
BCF (Hz)	3×10^{-2} *	4×10^{-1} NS	4×10^{-5} ***	4×10^{-10} ***	3×10^{-6} ***	3×10^{-7} ***	4×10^{-18} ***	1×10^{-28} ***	1×10^{-19} ***	1×10^{-9} ***	8×10^{-23} ***	4×10^{-12} ***	7×10^{-6} ***	2×10^{-1} NS	2×10^{-3} **
Angle of attack (°)	2×10^{-50} ***	2×10^{-67} ***	1×10^{-68} ***	2×10^{-71} ***	8×10^{-66} ***	5×10^{-28} ***	2×10^{-37} ***	3×10^{-39} ***	3×10^{-35} ***	4×10^{-13} ***	5×10^{-14} ***	1×10^{-12} ***	6×10^{-1} NS	5×10^{-1} NS	3×10^{-1} NS
Percentage of attack (%)	2×10^{-13} ***	1×10^{-24} ***	3×10^{-36} ***	7×10^{-53} ***	2×10^{-56} ***	2×10^{-12} ***	6×10^{-26} ***	3×10^{-43} ***	5×10^{-50} ***	1×10^{-8} ***	7×10^{-25} ***	2×10^{-36} ***	2×10^{-10} ***	4×10^{-25} ***	2×10^{-13} ***
Percentage of parallel (%)	1×10^{-13} ***	2×10^{-17} ***	5×10^{-19} ***	1×10^{-30} ***	5×10^{-36} ***	3×10^{-6} ***	2×10^{-9} ***	5×10^{-18} ***	4×10^{-26} ***	6×10^{-2} NS	2×10^{-5} ***	2×10^{-13} ***	3×10^{-2} *	3×10^{-8} ***	2×10^{-5} ***
Percentage of departure (%)	3×10^{-11} ***	4×10^{-18} ***	8×10^{-28} ***	4×10^{-44} ***	1×10^{-45} ***	4×10^{-11} ***	8×10^{-23} ***	1×10^{-39} ***	6×10^{-43} ***	4×10^{-8} ***	5×10^{-23} ***	3×10^{-31} ***	1×10^{-10} ***	2×10^{-19} ***	4×10^{-9} ***
Crossover frequency (Hz)	2×10^{-13} ***	2×10^{-23} ***	5×10^{-36} ***	7×10^{-53} ***	1×10^{-50} ***	2×10^{-11} ***	4×10^{-26} ***	3×10^{-43} ***	2×10^{-44} ***	2×10^{-9} ***	1×10^{-25} ***	1×10^{-31} ***	2×10^{-10} ***	2×10^{-19} ***	9×10^{-8} ***

Supplementary Note 1

Calculation for the Laplace Pressure. A pressure drop was calculated for different size sessile droplets. This Laplace pressure jump across the interface is directly proportional to the surface tension between the continuous and dispersed phases[1]:

$$\Delta P = 2\gamma/R \tag{1}$$

where, γ and R are the interfacial tension between the two phases and the radius of the droplet, respectively. Assuming the smallest and largest droplets with a size of 30 µm and 140 µm in radius, and using a surface tension of 18.5 mN/m between biocompatible oil (3M^{TM} Novec^{TM}) with 2% of a biocompatible surfactant as the continuous phase and sperm and buffer solution as the dispersed phases[2], the maximum change in the pressure domain inside the droplet encapsulating the sperm as a result of changing in the droplet size is:

$$\Delta P_{\text{max}} = 2\gamma\left(\frac{1}{R_{\text{smallest droplet}}} - \frac{1}{R_{\text{largest droplet}}}\right) = 0.96\%\ P_{\text{atmosphere}} \tag{2}$$

Supplementary Note 2

Theoretical Model for the Angle of Attack. A theoretical model was developed based on similarity of triangles to obtain the angle of attack, α, for sperm of length l with a beating amplitude of δ at a curved interface with a radius of curvature of R (Supplementary Fig. 17).

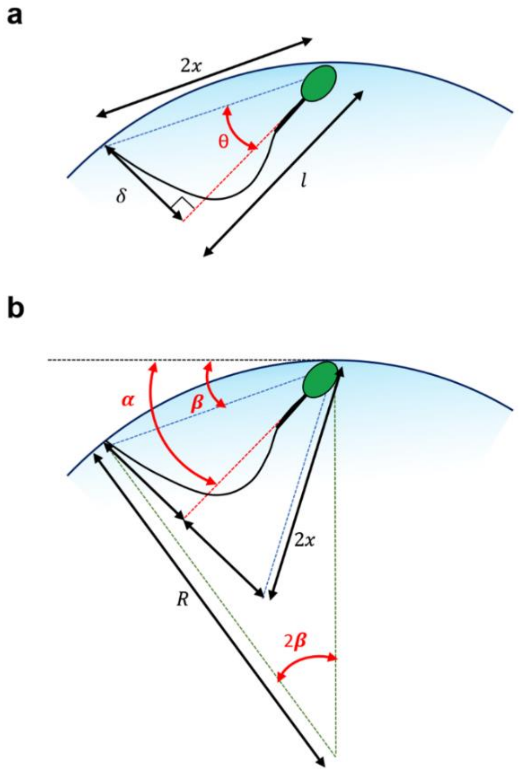

Supplementary Figure 17 | Theoretical model for calculating the angle of attack at curvatures. (**a**) Sperm of length l with the conical envelope of the flagellar wave aligned with the curvature and (**b**) the schematic used to drive the theoretical model for the angle of attack, α, based on the flagellar wave amplitude, δ, and the radius of curvature, R. θ is the angle between the longitudinal axis of sperm and the edge of the flagellar envelope, β is the angle between the edge of the flagellar envelope and the tangent line to the droplet, and $2x$ is the hypothenuse of the right-angle triangle formed by δ and l.

The hypothenuse of the right-angle triangle formed by δ and l in Supplementary Fig. 17 is identified as $2x$ and can be described as:

$$2x = \sqrt{\delta^2 + l^2} \tag{3}$$

$$x = \frac{\sqrt{\delta^2 + l^2}}{2} \tag{4}$$

The angle between the longitudinal axis of sperm and the edge of the flagellar envelope, θ, is:

$$\tan\theta = \tan^{-1}(\delta/l) \tag{5}$$

Therefore, the angle between the radii of the curvature formed by the conical envelope, 2β, is:

$$\beta = \sin^{-1}(x/R) \tag{6}$$

$$\sin\beta = \frac{\sqrt{\delta^2 + l^2}}{2R} \tag{7}$$

Since $\alpha = \beta + \theta$, the angle of attack can be obtained as a function of δ, l, and R as:

$$\alpha = \sin^{-1}\frac{\sqrt{\delta^2 + l^2}}{2R} + \tan^{-1}\left(\frac{\delta}{l}\right) \tag{8}$$

References

1 Pellicer, J., Garcia-Morales, V. & Hernandez, M. On the demonstration of the Young-Laplace equation in introductory physics courses. *Physics Education* **35**, 126-129 (2000).

2 Raveshi, M. R., Agnihotri, S. N., Sesen, M., Bhardwaj, R. & Neild, A. Selective droplet splitting using single layer microfluidic valves. *Sens. Actuators B Chem.* **292**, 233-240 (2019).
