# Supplementary material for: Curvature in the reproductive tract alters sperm–surface interactions
Source: Nat Commun. 2021 Jun 8;12:3446. doi: 10.1038/s41467-021-23773-x (PMC8187733; doi:10.1038/s41467-021-23773-x)
Supplement: Supplementary file 2 — Description of Additional Supplementary Files [file 41467_2021_23773_MOESM2_ESM.docx]

Description for Additional Supplementary Files

Title: Supplementary Movie 1

Description: Compilation comparing surface-attacking sperm motility mode with transition and progressive motility modes. (a) Aggressive attacking mode for sperm in a 40-µm radius droplet, (b) transition mode for sperm in a 73-µm radius droplet, and (c) progressive surfaces aligned motility mode for sperm in a 110-µm radius droplet. All videos are in real time, and scale bars, 50 µm. Images were contrast-adjusted for clarity.

Title: Supplementary Movie 2

Description: Aggressive attacking mode for sperm in a 40-µm radius droplet. The video is in real time, and scale bars, 50 µm. Images were contrastadjusted for clarity.

Title: Supplementary Movie 3

Description: Compass-like behaviour of sperm in a 35-µm radius droplet. The video is in real time, and scale bars, 20 µm. Images were contrastadjusted for clarity.

Title: Supplementary Movie 4

Description: Transition mode for sperm in a 73-µm radius droplet. The video is in real time, and scale bars, 50 µm. Images were contrast-adjusted for clarity.

Title: Supplementary Movie 5

Description: Progressive surfaces aligned motility mode for sperm in a 110-µm radius droplet. The video is in real time, and scale bars, 50 µm. Images were contrast-adjusted for clarity.

Title: Supplementary Movie 6

Description: Progressive surface aligned motility mode in a 110 µm-radius droplet for up to 56 s. The video is in real time, and scale bar, 50 µm. Images were contrast-adjusted for clarity.

Title: Supplementary Movie 7

Description: Compilation comparing sperm swimming behaviours at soft liquid-liquid curvatures inside microchannels. (a) Head-on swimming in the attacking mode, and boundary following behavior in (b) transition and (c) progressive modes. All videos are in real time and scale bars, 100 µm. Images were contrast-adjusted for clarity.
