# Supplementary material for: Curvature in the reproductive tract alters sperm–surface interactions
Source: Nat Commun. 2021 Jun 8;12:3446. doi: 10.1038/s41467-021-23773-x (PMC8187733; doi:10.1038/s41467-021-23773-x)
Supplement: Supplementary file 10 — Reporting Summary [file 41467_2021_23773_MOESM10_ESM.pdf]

nature research

Corresponding author(s): Dr Reza Nosrati and Prof Adrian Neild

Last updated by author(s): Apr 29, 2021

Reporting Summary

Nature Research wishes to improve the reproducibility of the work that we publish. This form provides structure for consistency and transparency in reporting. For further information on Nature Research policies, see our Editorial Policies and the Editorial Policy Checklist.

Statistics

For all statistical analyses, confirm that the following items are present in the figure legend, table legend, main text, or Methods section.

n/a	Confirmed	
☐	☒	The exact sample size (n) for each experimental group/condition, given as a discrete number and unit of measurement
☐	☒	A statement on whether measurements were taken from distinct samples or whether the same sample was measured repeatedly
☐	☒	The statistical test(s) used AND whether they are one- or two-sided<br>*Only common tests should be described solely by name; describe more complex techniques in the Methods section.*
☒	☐	A description of all covariates tested
☒	☐	A description of any assumptions or corrections, such as tests of normality and adjustment for multiple comparisons
☐	☒	A full description of the statistical parameters including central tendency (e.g. means) or other basic estimates (e.g. regression coefficient) AND variation (e.g. standard deviation) or associated estimates of uncertainty (e.g. confidence intervals)
☐	☒	For null hypothesis testing, the test statistic (e.g. F, t, r) with confidence intervals, effect sizes, degrees of freedom and P value noted<br>*Give P values as exact values whenever suitable.*
☒	☐	For Bayesian analysis, information on the choice of priors and Markov chain Monte Carlo settings
☒	☐	For hierarchical and complex designs, identification of the appropriate level for tests and full reporting of outcomes
☐	☒	Estimates of effect sizes (e.g. Cohen's d, Pearson's r), indicating how they were calculated

Our web collection on statistics for biologists contains articles on many of the points above.

Software and code

Policy information about availability of computer code

Data collection	Olympus cellSens Dimension 2.1 - MAESFLO™ 3.3.1 software (Microfluidic Flow Control software provided by Fluigent company)
Data analysis	MATLAB R2019b (custom code) - ImageJ version 1.52v - Microsoft Excel 2016 - AutoCAD 2020

For manuscripts utilizing custom algorithms or software that are central to the research but not yet described in published literature, software must be made available to editors and reviewers. We strongly encourage code deposition in a community repository (e.g. GitHub). See the Nature Research guidelines for submitting code & software for further information.

Data

Policy information about availability of data

All manuscripts must include a data availability statement. This statement should provide the following information, where applicable:

- Accession codes, unique identifiers, or web links for publicly available datasets
- A list of figures that have associated raw data
- A description of any restrictions on data availability

All microscopy images and relevant data supporting the findings of this study are available within the article and its Supplementary Information files or upon request from the corresponding author, Reza Nosrati, at reza.nosrati@monash.edu . Source data are provided with this paper.
The female fallopian tube cross-section image in Figure 1a was adopted without change from https://www.flickr.com/photos/euthman/2760474960/ under CC BY 2.0.

Field-specific reporting

Please select the one below that is the best fit for your research. If you are not sure, read the appropriate sections before making your selection.

[x] Life sciences [ ] Behavioural & social sciences [ ] Ecological, evolutionary & environmental sciences

For a reference copy of the document with all sections, see nature.com/documents/nr-reporting-summary-flat.pdf

Life sciences study design

All studies must disclose on these points even when the disclosure is negative.

Sample size

Observation was made using at least 45 droplets of each size with 300 data points per observation (n=45, 46, 45, 46, 45 and 45 biologically independent cells examined over 5, 4, 4, 4, 5 and 4 independent experiments for sperm swimming in droplets ranging in radius from 36-40 µm, 50-60 µm, 70-80 µm, 90-100 µm, 110-120 µm and 130-140 µm, respectively).
Sample size was determined based on power calculations. Based on preliminary results for 10 samples per data point (n=10), the minimum effect size that we were capturing as statistically significant was 0.24 (reported for the change in the crossover frequency in Fig. 3b and Supplementary Table 3). Based on statistical theory for the model used here, to detect this effect size of 0.24, at the significance level of 0.05 and with an 80% chance (the accepted power), we need at least 39 samples per data point. Therefore, our current sample sizes of 45 and 46 achieves the required level of specificity and sensitivity to capture this change.

Data exclusions

No data were excluded from the analysis.

Replication

All attempts at replication were successful (at least 4 independent biological experiments and at least 4 different bulls). Each experiment was repeated with droplets produced in at least three different chips, in different days, and with sperm from different bulls, with no identifiable difference in the experimental outcomes. For sperm swimming in droplets ranging in radius from 36-40 µm, 50-60 µm, 70-80 µm, 90-100 µm, 110-120 µm and 130-140 µm, we analyzed n=45, 46, 45, 46, 45 and 45 biologically independent sperm cells over 5, 4, 4, 4, 5 and 4 independent experiments , respectively.

Randomization

Bulls were selected randomly and sperm from at least 4 independent bulls were used in the experiments (~10 cells per bull).

Blinding

Experiments and analysis were performed by 3 different persons (first, second and third authors) and with sperm from different unidentified bulls to ensure blinded experiments and evaluation. Also, during the experiments, the samples and bull types were anonymous to the person who was running the experiment.

Reporting for specific materials, systems and methods

We require information from authors about some types of materials, experimental systems and methods used in many studies. Here, indicate whether each material, system or method listed is relevant to your study. If you are not sure if a list item applies to your research, read the appropriate section before selecting a response.

Materials & experimental systems

n/a	Involved in the study	
[ ]	[x]	Antibodies
[x]	[ ]	Eukaryotic cell lines
[x]	[ ]	Palaeontology and archaeology
[x]	[ ]	Animals and other organisms
[x]	[ ]	Human research participants
[x]	[ ]	Clinical data
[x]	[ ]	Dual use research of concern

Methods

n/a	Involved in the study	
[x]	[ ]	ChIP-seq
[x]	[ ]	Flow cytometry
[x]	[ ]	MRI-based neuroimaging

Antibodies

Antibodies used

Anti-Phosphotyrosine Antibody, clone 4G10 (05321, Sigma-Aldrich)

Validation

Anti-Phosphotyrosine Antibody, clone 4G10 detects tyrosine phosphorylated proteins in all species. This unique monoclonal antibody is validated for use in IC, IH, IP, WB and is backed by hundreds of publications as it is mentioned in the provider website:
https://www.merckmillipore.com/AU/en/product/Anti-Phosphotyrosine-Antibody-clone-4G10,MM_NF-05-321
